# Natural killer cell-intrinsic type I IFN signaling controls *Klebsiella pneumoniae* growth during lung infection

**DOI:** 10.1371/journal.ppat.1006696

**Published:** 2017-11-07

**Authors:** Masa Ivin, Amy Dumigan, Filipe N. de Vasconcelos, Florian Ebner, Martina Borroni, Anoop Kavirayani, Kornelia N. Przybyszewska, Rebecca J. Ingram, Stefan Lienenklaus, Ulrich Kalinke, Dagmar Stoiber, Jose A. Bengoechea, Pavel Kovarik

**Affiliations:** 1 Max F. Perutz Laboratories, University of Vienna, Vienna Biocenter (VBC), Vienna, Austria; 2 Wellcome-Wolfson Institute for Experimental Medicine, Queen's University Belfast, Belfast, United Kingdom; 3 Vienna Biocenter Core Facilities, Histopathology Facility, Dr. Bohr-Gasse 3, Vienna, Austria; 4 Institute for Experimental Infection Research, TWINCORE, Centre for Experimental and Clinical Infection Research, a joint venture between the Hannover Medical School and the Helmholtz Centre for Infection Research, Hannover, Germany; 5 Institute of Pharmacology, Medical University of Vienna, Vienna, Austria; 6 Ludwig Boltzmann Institute for Cancer Research, Vienna, Austria; University of California Davis School of Medicine, UNITED STATES

## Abstract

*Klebsiella pneumoniae* is a significant cause of nosocomial pneumonia and an alarming pathogen owing to the recent isolation of multidrug resistant strains. Understanding of immune responses orchestrating *K*. *pneumoniae* clearance by the host is of utmost importance. Here we show that type I interferon (IFN) signaling protects against lung infection with *K*. *pneumoniae* by launching bacterial growth-controlling interactions between alveolar macrophages and natural killer (NK) cells. Type I IFNs are important but disparate and incompletely understood regulators of defense against bacterial infections. Type I IFN receptor 1 (*Ifnar1*)-deficient mice infected with *K*. *pneumoniae* failed to activate NK cell-derived IFN-γ production. IFN-γ was required for bactericidal action and the production of the NK cell response-amplifying IL-12 and CXCL10 by alveolar macrophages. Bacterial clearance and NK cell IFN-γ were rescued in *Ifnar1*-deficient hosts by *Ifnar1*-proficient NK cells. Consistently, type I IFN signaling in myeloid cells including alveolar macrophages, monocytes and neutrophils was dispensable for host defense and IFN-γ activation. The failure of *Ifnar1*-deficient hosts to initiate a defense-promoting crosstalk between alveolar macrophages and NK cell was circumvented by administration of exogenous IFN-γ which restored endogenous IFN-γ production and restricted bacterial growth. These data identify NK cell-intrinsic type I IFN signaling as essential driver of *K*. *pneumoniae* clearance, and reveal specific targets for future therapeutic exploitations.

## Author summary

The isolation of multidrug-resistant *Klebsiella pneumoniae* strains has significantly narrowed, or in some settings completely removed, the therapeutic options for the treatment of *Klebsiella* infections. Therapies targeting the immune system rather than the pathogen represent important alternatives. Despite the clinical relevance, there are still major gaps in our understanding of immune responses which drive the clearance of this pathogen. Type I interferons (IFNs) are known as powerful immune system regulators yet their effects on bacterial infections are disparate and remain elusive. In this study we show that type I IFN signaling is indispensable for mounting a protective and bacterial clearance-promoting immune response against *K*. *pneumoniae*. *K*. *pneumoniae*-induced type I IFNs launch a crosstalk between alveolar macrophages and NK cells by enabling NK cell IFN-γ production which in turn activates the macrophage anti-microbial armament. Type I IFN-responsive NK cells or IFN-γ administration rescue *K*. *pneumoniae* clearance in type I IFN-unresponsive hosts. Our study suggests that manipulation of type I IFN or IFN-γ levels might represent a valid strategy for treatment of drug-resistant *K*. *pneumoniae* infections.

## Introduction

*Klebsiella pneumoniae* is a capsulated Gram negative pathogen which causes a wide range of infectious diseases, from urinary tract infections to pneumonia, the latter being particularly devastating among immunocompromised patients [1]. Of particular concern is the increasing isolation of multidrug resistant strains that narrows the therapeutic options for the treatment of *K*. *pneumoniae* infections [2–4]. To further complicate this scenario, recent population genomic studies have shown that virulent and multidrug resistant clones have access to a diverse mobile pool of virulence and antimicrobial resistance genes [2, 5] hence making possible the emergence of an extremely drug-resistant hypervirulent *K*. *pneumoniae* strain capable of causing untreatable infections in healthy individuals. It is then not surprising that multidrug resistant *K*. *pneumoniae* has been singled out as a significant threat to global public health by the World Health Organization, Centers of Diseases Control and Prevention, European Union and other organizations [4]. In light of this growing health problem, it is essential to better understand the immune pathways that are critical to host defense towards *K*. *pneumoniae*.

Successful defense against infections requires a coordinated action of multiple immune cell subsets. In this context, it is widely appreciated that type I interferons (IFNs) decisively coordinate immune responses by modulating cell-autonomous immunity and inflammatory responses, and by dictating immune cell-to-cell communications [6, 7]. While type I IFNs are the major effector cytokines of the host defense response against viral infections, a body of mainly recent data indicate that type I IFNs are also produced in response to bacteria [8–10]. However, depending on the bacterial infection, type I IFNs exert seemingly opposing functions [8, 10]. Type I IFNs protect against the progression of a localized *Streptococcus pneumoniae* lung infection to invasive disease [11, 12], prevent IL-1β-mediated tissue-damaging hyperinflammation in invasive soft tissue infection with *Streptococcus pyogenes* [13, 14] and restrict the growth of *Legionella pneumophila* in macrophages [15]. By contrast, type I IFNs impair the clearance of the intracellular pathogen *Mycobacterium tuberculosis* [16–18] and they are detrimental to host survival after *Francisella tularensis* infection [19, 20]. Overall, it is currently impossible to predict from the pathogen tropism or biology whether type I IFNs will be beneficial or detrimental for the host. This knowledge gap calls for additional mechanistic studies dissecting the type I IFN functions in other bacterial infections in order to define common and unique principles of the role of type I IFNs in host defense against bacterial infections.

Research over the last twenty years demonstrates that activation of early inflammatory responses, including production of TNF, IL-12, IL-23, IL-17 and IFN-γ, is essential to clear *K*. *pneumoniae* infections [21–23]. The role of type I IFNs in *K*. *pneumoniae* has so far not been addressed. Here we show that type I IFN signaling coordinates the communication between macrophages and NK cells to launch a protective immune response during lung infection with *K*. *pneumoniae*. Type I IFNs produced by macrophages upon *K*. *pneumoniae* challenge promote IFN-γ production by NK cells. IFN-γ in turn feeds back to prime macrophages for enhanced IL-12 production and bacterial killing. These results establish that NK cell-restricted type I IFN signaling entails resistance against severe lung infection caused by *K*. *pneumoniae*.

## Results

### Type I IFN signaling-deficient mice are susceptible to *K*. *pneumoniae* infection

**Fig 1:**
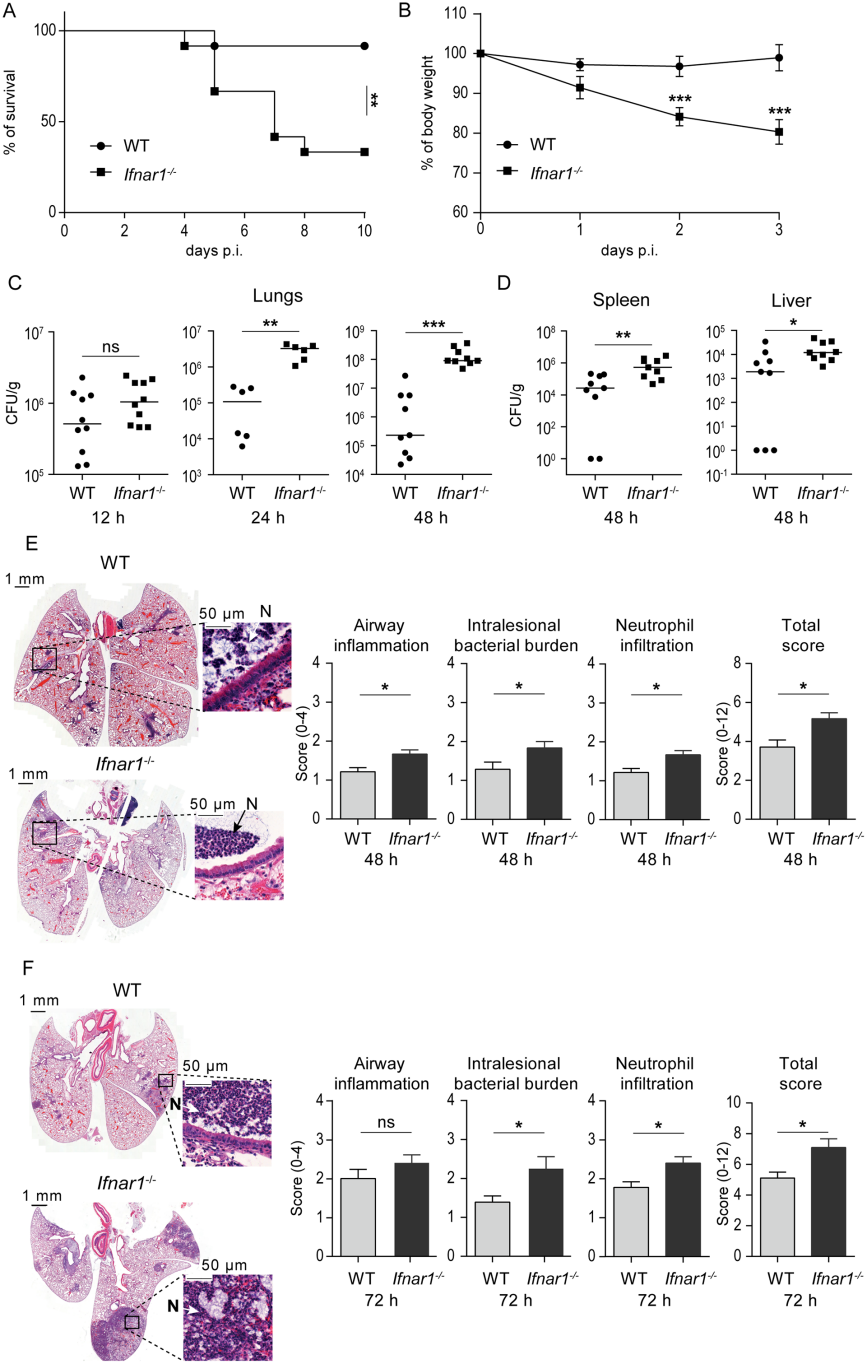
Type I IFN signaling protects against *K*. *pneumoniae* lung infection by controlling bacterial growth and lung pathology. **(A**) WT and *Ifnar1*^-/-^ mice (n = 12 per genotype) were infected intranasally (5 x 10^4^ CFU of *K*. *pneumoniae*), and survival was monitored for 10 days. Kaplan-Meier survival curves are shown. Statistical evaluation: Log-rank (Mantel-Cox) test. **, P < 0.01. (**B**) Weight changes during the first 3 days following infection. Data are represented as mean ± SEM. Statistical evaluation: unpaired Student’s *t* test. ***, P < 0.001. (**C, D**) WT and *Ifnar1*^-/-^ mice were infected as in (A) and bacterial loads were determined in lungs 12, 24 and 48 h p. i. (C), and in spleens and livers 48 h p. i. (D). Bacterial load is presented as CFU per g of analyzed organ per infected animal. Dot plots: horizontal bars represent median. Statistical evaluation: Mann-Whitney test. *, P < 0.05; **, P < 0.01; ***, P < 0.001; ns, not significant. **(E, F)** H&E-stained sections of lungs of infected WT and *Ifnar1*^-/-^ animals at 48 (E) and 72 h (F) p. i., together with quantification of airway inflammation, intralesional bacterial burden, neutrophilic infiltration and the combined histopathology analysis (total histopathology score) (n = 7, 6, 9 and 10 mice for WT 48 h p. i., *Ifnar1*^-/-^ 48 h p. i., WT 72 h p. i., *Ifnar1*^-/-^ 72 h p. i., respectively). Insets: Arrows and N indicate neutrophils. Note an increased airway inflammation, intralesional bacterial burden and neutrophilic infiltration in lungs from *Ifnar1*^-/-^ animals. Histopathology scores were determined by blinded scoring; error bars, mean ± SEM. Statistical evaluation: Mann-Whitney test. *, P < 0.05; ns, not significant.

The importance of type I IFN signaling during *K*. *pneumoniae*-induced pneumonia was assessed by infecting type I IFN 1 receptor-deficient (*Ifnar1*^*-/-*^) mice. Ifnar1 is one of the subunits of the type I IFN receptor which mediates type I IFN responses in innate and acquired immunity to infection. Mice were intranasally infected with *K*. *pneumoniae* strain 52.145. This infection model recapitulates *Klebsiella*-triggered human pneumonia [24, 25]. Strain 52.145 belongs to the *K*. *pneumoniae* KpI group which includes the vast majority of strains most frequently associated with human infection, including numerous multidrug-resistant or hypervirulent clones [2]. This strain encodes all virulence functions significantly associated with invasive community-acquired disease in humans [2, 5]. *Ifnar1*^*-/-*^ mice infected with 5 x 10^4^ CFU *K*. *pneumoniae* 52.145 exhibited a markedly decreased survival (Fig 1A) and increased weight loss (Fig 1B) as compared to wild-type (WT) controls, demonstrating essential contribution of type I IFN signaling to host defense against this pathogen.

To elucidate possible mechanisms by which the absence of type I IFN signaling resulted in increased lethality, we examined the ability of *Ifnar1*^*-/-*^ mice to control bacterial growth. Analysis of bacterial loads in lungs 12, 24 and 48 h following *K*. *pneumoniae* infection revealed a significant defect of *Ifnar1*^*-/-*^ mice to restrict bacterial replication (Fig 1C). Both *Ifnar1*^*-/-*^ and WT mice exhibited similar bacterial burdens 12 h post infection (p.i.) (Fig 1C, left panel). However, in contrast to WT mice, *Ifnar1*^*-/-*^ mice failed to control bacterial growth at later time points resulting in significantly higher bacterial burdens in lungs at 24 and 48 h post infection (p.i.) (Fig 1C, middle and right panel). Consistently, the difference in CFU between *Ifnar1*^*-/-*^ and WT mice was increasing with time of infection. Higher bacterial loads were also found in the spleen and liver of *Ifnar1*^*-/-*^ mice than in organs of WT animals at 48 h p.i. (Fig 1D). The bacterial burden in the spleen and liver was negligible and similar in both genotypes at 12 h p.i. Analysis of lung sections 48 and 72 h p.i. showed more severe bronchopneumonia in *Ifnar1*^*-/-*^ mice compared to WT controls, as revealed by a higher overall histopathology score comprising airway inflammation, intralesional bacterial burden and neutrophil infiltration (Fig 1E and 1F). The bronchopneumonia involved bronchi, bronchioles and to a lesser extent alveolar ducts and alveoli. The inflammation was predominantly neutrophilic, and aggregates of bacteria were evident within the foci of inflammation. No appreciable differences between *Ifnar1*^*-/-*^ and WT mice were noted in lung histology of animals which were given intranasal PBS (S1A Fig).

The lung pathology analysis suggested that severe lung destruction was the cause of increased mortality of *Ifnar1*^*-/-*^ animals. To test this, we infected a cohort of *Ifnar1*^*-/-*^ mice and monitored the progress of bronchopneumonia toward a lethal disease by comparing lungs of mice which were reaching behavioral and/or patho-physiological humane endpoints with lungs of mice which were showing mild symptoms at the same time of infection. In addition, we sampled lungs of mice which survived longer than 10 days and appeared recovered. Lung section analyses revealed that animals showing the most severe symptoms, i.e. animals approaching human endpoints, exhibited large areas of lung inflammation (more than 60% of the lung tissue affected) including increased intralesional bacteria and neutrophil infiltration (S1B Fig, left panel). The high degree of pathological changes in lungs of these mice was not compatible with life since animals with similarly severe symptoms ultimately developed a lethal disease in survival experiments (Fig 1). In contrast, animals showing only mild symptoms or animals surviving the 10 day observation period exhibited low or no lung inflammation at all, respectively (S1B Fig, middle and right panels).

### *K*. *pneumoniae* induces type I IFN in IRF3- and TLR4-dependent manner

**Fig 2:**
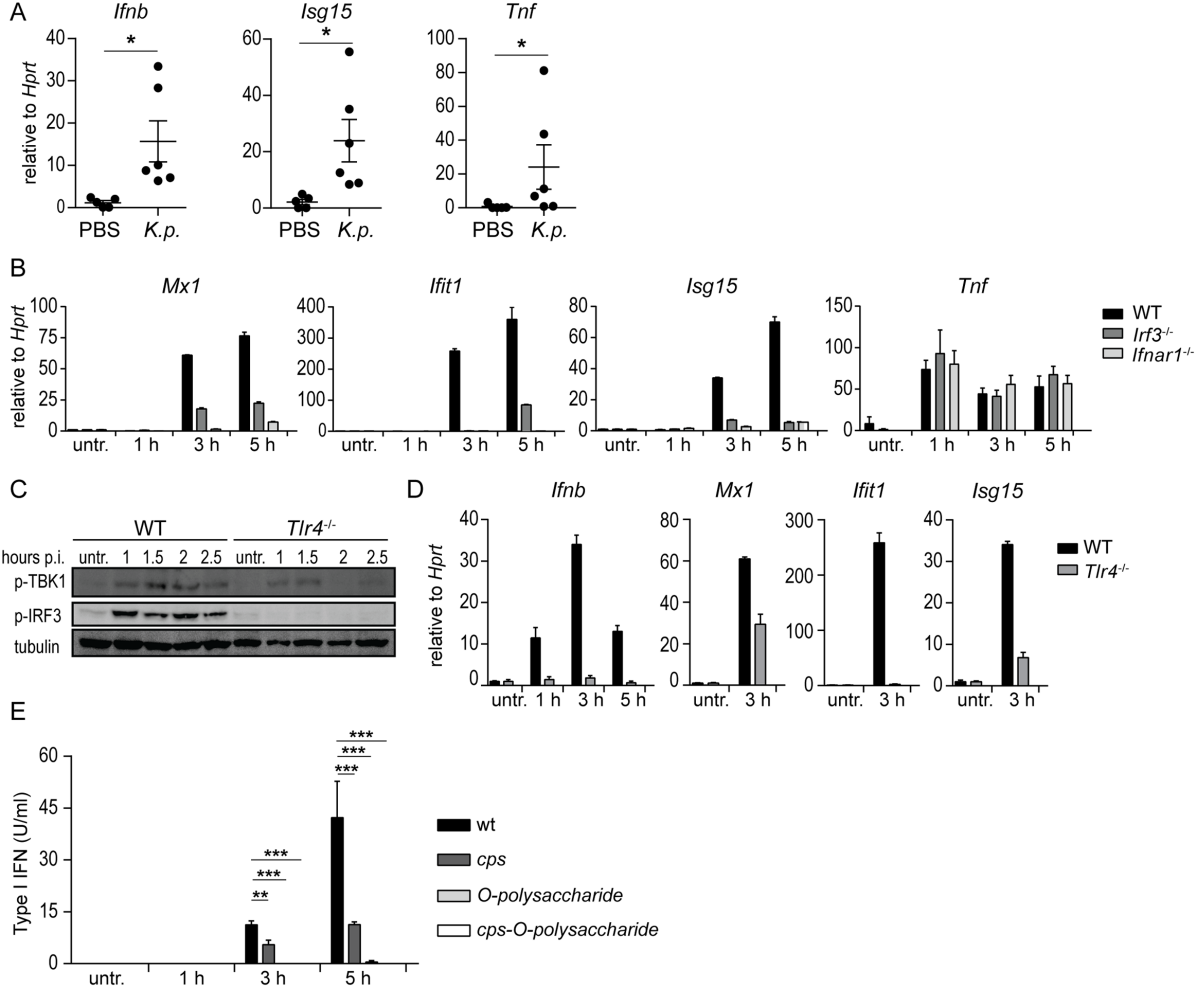
Induction of type I IFN signaling in macrophages by *K*. *pneumoniae* is dependent on *Irf3* and *Tlr4*, and the bacterial capsule polysaccharide (CPS) and LPS O-polysaccharide. **(A)** Mouse alveolar macrophages isolated from *K*. *pneumoniae*-infected (intranasal, 5 x 10^4^ CFU) or PBS-treated WT mice were analyzed for expression of *Ifnb*, *Isg15* and *Tnf* using qPCR 24 h p.i. (n = 5, PBS; n = 6, infection). **(B)** BMDMs from WT, *Irf3*^-/-^ and *Ifnar1*^-/-^ mice were left untreated or infected for indicated time points with *K*. *pneumoniae* (MOI = 70), and mRNA levels of *Mx1*, *Ifit1*, *Isg15* and *Tnf* were determined by qPCR. Error bars, mean ± SEM (n > 3). (**C, D**) WT and *Tlr4*^-/-^ BMDMs were infected as in (B) for indicated time points, or left untreated. Phospho-TBK1 (p-TBK1), phospho-IRF3 (p-IRF3) and tubulin (loading control) were detected in whole cell extracts by Western blotting (E), and *Ifnb*, *Mx1*, *Ifit1* and *Isg15* mRNA levels were quantitated by qPCR (D). **(E)** BMDMs were infected as in (B) with a *cps*, O-polysaccharide, and double *cps*-O-polysaccharide *K*. *pneumoniae* mutants for indicated time points, or left untreated, and type I IFN levels in the supernatants were quantitated using bioassays. Statistical evaluation in (A) and (E): unpaired Student’s *t* test; error bars, mean ± SEM (n > 3); *, P < 0.05; **, P < 0.01; ***, P < 0.001.

Having established the importance of type I IFN signaling for host defense against *K*. *pneumoniae*, we sought to determine the signaling pathway(s) activated by the pathogen to induce type I IFN production and signaling. Myeloid cells such as alveolar macrophages produce type I IFNs in response to many bacterial pathogens [8–10]. To test whether alveolar macrophages are type I IFN producers during *K*. *pneumoniae* infection, alveolar macrophages (CD45^+^CD11c^high^SiglecF^+^) were isolated from infected C57Bl/6 mice 24 h p.i. These cells displayed induction of *Ifnb*, the key type I IFN gene, when compared to uninfected controls (Fig 2A). Consistently, the type I IFN-stimulated gene (ISG) *Isg15* was also induced (Fig 2A). Induction of *Tnf* confirmed that alveolar macrophages were activated by the infection (Fig 2A). Type I IFNs were also induced in the mouse alveolar macrophage cell line MH-S and bone marrow derived macrophages (BMDMs) infected with *K*. *pneumoniae* (S2A Fig). In agreement, *K*. *pneumoniae* infection induced the expression of the ISGs *Mx1*, *Ifit1* and *Isg15* in BMDMs (Fig 2B). *Tnf* expression was similar in WT and *ifnar1*^*-/-*^ BMDMs (Fig 2B). Further, we observed ISG15 modification (ISGylation) of proteins in *K*. *pneumoniae*-infected cells (S2B Fig). The induction of ISGs and ISGylation by *K*. *pneumoniae* was abrogated in *Ifnar1*^*-/-*^ BMDMs demonstrating the requirement for type I IFN signaling (S2B Fig).

The central tenet of type I IFN production is that the initial wave of type I IFN production relies on the activation of the IFN-regulatory factor (IRF) 3 [26]. *K*. *pneumoniae*-induced expressions of *Ifnb* and of the ISGs *Mx1*, *Ifit1* and *Isg15* were ablated in infected *Irf3*^*-/-*^ BMDMs Fig 2B). In contrast, *Tnf* expression was not affected by the *Irf3* deletion (Fig 2B). These data reveal a fundamental role of IRF3 in induction of *K*. *pneumoniae*-triggered type I IFNs and ISGs.

Bacteria trigger type I IFN production by activation of various pattern recognition receptors (PRRs) [8, 10]. In vivo and in vitro evidence has demonstrated that TLR4 governs host defenses against *K*. *pneumoniae* [27–30]. To test the involvement of TLR4 in *K*. *pneumoniae*-induced IFN-β production, we employed BMDMs derived from *Tlr4*^*-/-*^ mice. Supporting the key role of TLR4 in *Ifnb* induction, *K*. *pneumoniae*-triggered phosphorylation of *Ifnb* gene drivers IRF3 and the IRF3 kinase TBK1 [26] were abrogated in *Tlr4*^*-/-*^ BMDMs (Fig 2C). The lack of TLR4 abolished induction of *Ifnb*, *Mx1*, *Ifit1* and *Isg15* and protein modification by ISG15 (ISGylation) (Fig 2D and S2C Fig). Consistent with the involvement of the canonical TLR4-activated pathway [26], macrophages lacking the adaptors TRIF and TRAM did not induce protein ISGylation and the expression of *Ifnb*, *Mx1*, *Ifit1* and *Isg15* in response to *K*. *pneumoniae* infection (S2D and S2E Fig). Phosphorylation of IRF3 and TBK1 was not stimulated in macrophages lacking the adaptors TRIF and TRAM, but proceeded normally in the absence of MyD88 (S2F Fig). Together, these results demonstrate that *K*. *pneumoniae*-induced type I IFN production and signaling is dependent on the TLR4-TRIF-TRAM-IRF3 pathway.

We and others have shown that *K*. *pneumoniae* capsule polysaccharide (CPS) and lipopolysaccharide (LPS) are recognized by TLR4 to launch inflammatory responses [31, 32]. Therefore, we hypothesized that these polysaccharides might be involved in TLR4-mediated type I IFN induction. To test this, macrophages were infected with a *cps*, O-polysaccharide, and double *cps*-O-polysaccharide *K*. *pneumoniae* mutants, and type I IFN was quantified in the supernatants of infected cells. The three mutants induced less type I IFN than the wild-type strain (Fig 2E) although the *cps* mutant induced more type I IFN than the two O-polysaccharide mutants. The lack of induction of type I IFN production was in agreement with the reduced phosphorylation of IRF3 triggered by the mutants (S2G Fig) indicating that the CPS and LPS O-polysaccharides are the *K*. *pneumoniae* factors activating TLR4 to induce type I IFN.

### Type I IFN signaling promotes IFN-γ, IL-12 and CXCL10 production in response to *K*. *pneumoniae* in vivo

**Fig 3:**
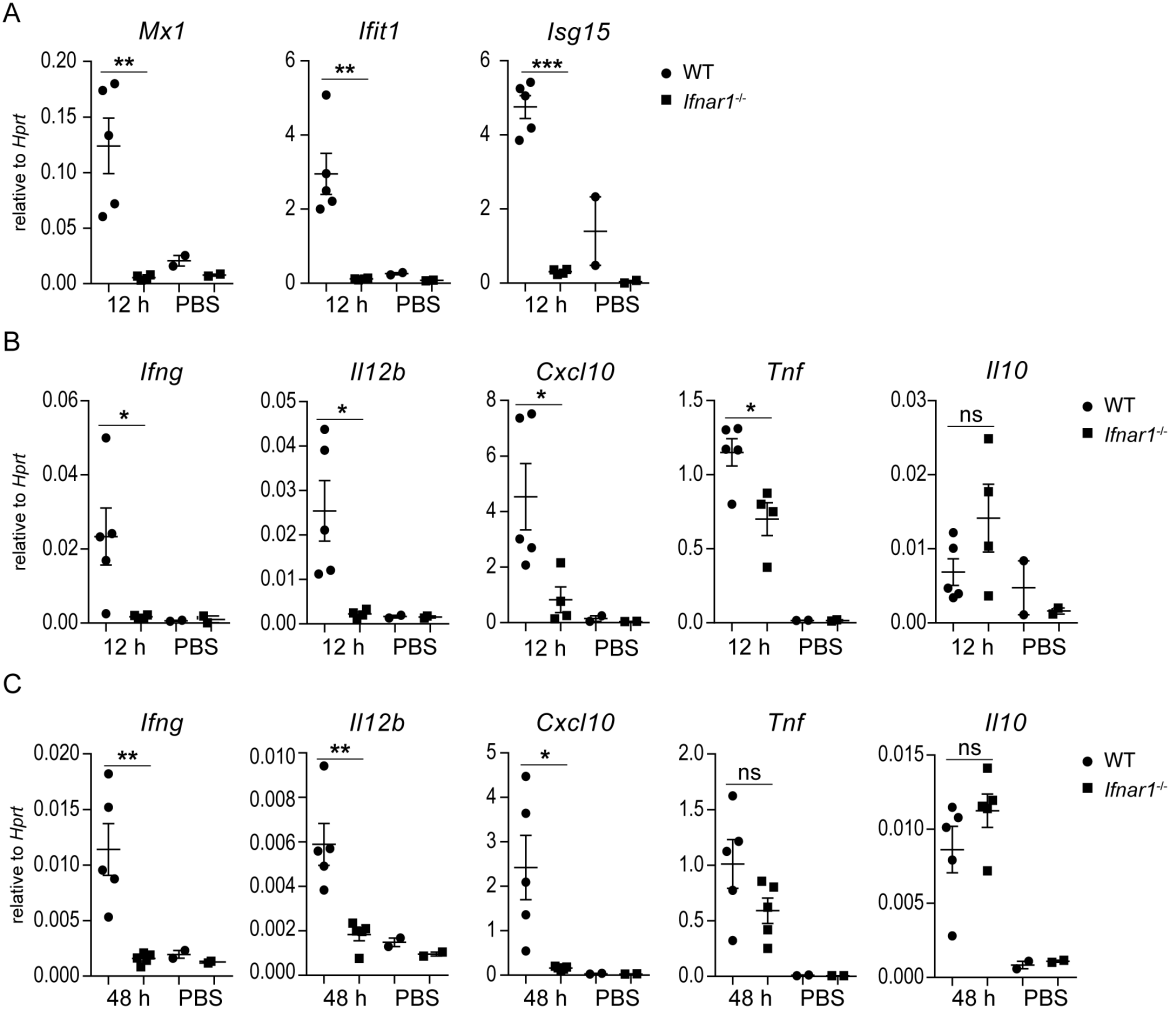
Type I IFN signaling is indispensable for IFN-γ, IL-12 and CXCL10 induction in *K*. *pneumoniae*-infected lungs. WT and *Ifnar1*^-/-^ mice were infected intranasally (5 x 10^4^ CFU of *K*. *pneumoniae*) for 12 **(A, B)** or 48 **(C)** h, or treated with PBS, and gene expression was determined by qPCR normalized to *Hprt*. (A) *Mx1*, *Ifit1* and *Isg15* mRNA levels in lungs. (B, C) *Ifng*, *Il12b*, *Cxcl10*, *Tnf* and *Il10* mRNA levels in lungs. Statistical evaluation: unpaired Student’s *t* test; error bars, mean ± SEM (n > 3); *, P < 0.05; **, P < 0.01; ***, P < 0.001; ns, not significant.

To confirm that type I IFN signaling is activated by *K*. *pneumoniae* also in vivo, we examined the lung tissue 12 h p.i. Expression of the ISGs *Mx1*, *Ifit1* and *Isg15* was induced in WT but not *Ifnar1*^*-/-*^mice (Fig 3A) corroborating the results obtained using infection of macrophages. To assess whether type I IFN signaling influenced the inflammatory response in lungs of *K*. *pneumoniae*-infected animals, we analyzed several inflammation-associated cytokines and chemokines 12 h p.i. *K*. *pneumoniae*–induced expression of IFN-γ, a critical cytokine for defense against lung infection with *K*. *pneumoniae* [22], was virtually absent in *Ifnar1*^*-/-*^ mice at both mRNA and protein levels (Fig 3B and S3A Fig). Similarly, the induction of IL-12 mRNA (*Il12b*) and protein (IL-12p70), a key IFN-γ inducer, was impaired in *Ifnar1*^*-/-*^ mice (Fig 3B and S3A Fig). The induction of CXCL10, a chemokine required for NK cell recruitment and host defense against *K*. *pneumoniae* [33], was abolished in *Ifnar1*^*-/-*^ mice (Fig 3B). The mRNA of the immediate early cytokine *Tnf* was induced upon infection in both WT and *Ifnar1*^*-/-*^ mice although the levels were lower in *Ifnar1*^*-/-*^ when compared to WT animals (Fig 3B). At the protein level, TNF was comparable in both genotypes (S3A Fig). Expression of the anti-inflammatory *Il10*, the neutrophil chemoattractant *Cxcl1* and the pro-inflammatory *Il1b*, were not affected by type I IFN signaling (Fig 3B and S3B Fig). The defect of *Ifnar1*^*-/-*^ mice in induction of *Ifng*, *Il12b* and *Cxcl10* was persistent and clearly detectable at 48 p.i. (Fig 3C). However, the expression of *Tnf* was no longer different between *Ifnar1*^*-/-*^ and WT mice at this later time point (Fig 3C). The expression of *Il10*, *Cxcl1* and *Il1b* was comparable in *Ifnar1*^*-/-*^ and WT mice at 48 h p.i. (Fig 3C and S3C Fig), as observed already at the 12 h time point (Fig 3B). Together, the lack of type I IFN signaling results in defect in the production of IFN-γ, IL-12 and CXCL10 in *K*. *pneumoniae*-infected lungs.

### Activation and accumulation of NK cells in *K*. *pneumoniae*-infected lungs is dependent on type I IFN signaling

**Fig 4:**
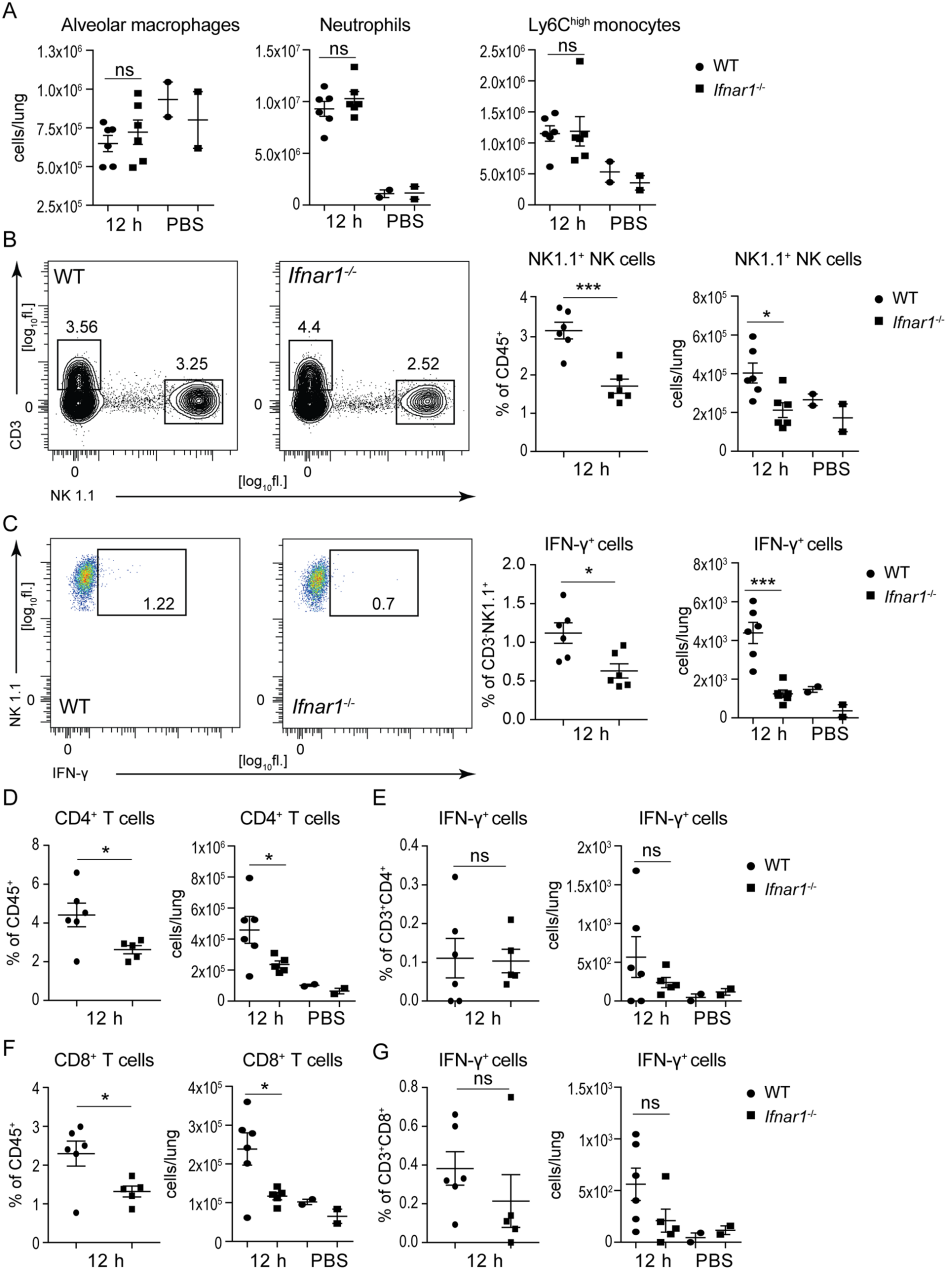
Type I IFN signaling induces NK cell accumulation and activation in lungs during *K*. *pneumoniae* infection. WT and *Ifnar1*^-/-^ mice were infected intranasally (5 x 10^4^ CFU of *K*. *pneumoniae*) for 12 h or treated with PBS and immune cell subsets in lungs were analyzed by flow cytometry. Cells were subgated for CD45^+^ cells. **(A)** Alveolar macrophage, neutrophil and inflammatory monocyte populations were detected as SiglecF^+^CD11c^high^, Cd11b^+^Ly6G^+^Ly6C^med^, and CD11b^+^Ly6G^-^Ly6C^high^, respectively. Populations are presented as total cells per lung calculated from percentages of individual subsets (shown in S4 Fig). **(B, C)** NK cells, detected as CD3^-^NK1.1^+^, and IFN-γ^+^ NK cells are shown. Representative plots of NK cells (B, left panels) and IFN-γ^+^ NK cells (C, left panels) are shown. Numbers indicate percentages in the outlined area of live, CD45^+^ cells. NK cell population in dot plots is shown both as percent of CD45^+^ cells as well as total cells per lung (B, right panels). IFN-γ^+^ cells are shown both as % of CD3^-^NK1.1^+^ cells and as total cells per lung (C, right panels). **(D-G)** CD4 T cells detected as CD3^+^CD4^+^ (D), IFN-γ-producing CD4 T cells detected as IFN-γ^+^ CD3^+^CD4^+^ (E), CD8 T cells detected as CD3^+^CD8^+^ (F), and IFN-γ-producing CD8 T cells detected as IFN-γ^+^ CD3^+^CD8^+^ (G) are shown in dot plots. CD4 and CD8 T cells are shown as percent of CD45^+^ cells (D and F, left panels) and as total cells per lung (D and F, right panels). IFN-γ^+^ CD4 and IFN-γ^+^ CD8 T cells are shown both as % of CD3^+^CD8^+^ cells (E and G, left panels) and as total cells per lung (E and G, right panels). Statistical evaluation: unpaired Student’s *t* test; error bars, mean ± SEM (n > 3); *, P < 0.05; **, P < 0.01; ***, P < 0.001; ns, not significant.

The failure of *Ifnar1*-deficient mice to induce IL-12 and IFN-γ upon *K*. *pneumoniae* infection suggested a defect in immune cells producing these cytokines. Flow cytometry analysis revealed comparable numbers of alveolar macrophages (4% CD11c^high^SiglecF^+^ alveolar macrophages of CD45^+^ leukocytes) in lungs of *Ifnar1*^-/-^ and WT animals 12 h after infection with *K*. *pneumoniae* (Fig 4A and S4A Fig). Infection did not increase the alveolar macrophage population over PBS-treated controls (Fig 4A). The numbers of neutrophils (Cd11b^+^Ly6G^+^Ly6C^med^) and inflammatory monocytes (CD11b^+^Ly6G^-^Ly6C^high^) in *K*. *pneumoniae*-infected lungs were also similar in both genotypes (Fig 4A, S4B Fig). Both neutrophils and inflammatory monocytes increased upon infection as compared to PBS-treated controls (Fig 4A), consistent with previous studies [21]. In contrast, the population of CD3^-^NK1.1^+^ NK cells, which have been implicated in defense against *K*. *pneumoniae* [34], was decreased in lungs of infected *Ifnar1*^-/-^ mice when compared to WT controls both in terms of percentage of total leukocytes as well as absolute cell numbers (Fig 4B). The NK cell numbers in infected *Ifnar1*^-/-^ mice were comparable to those in PBS-treated mice (Fig 4B). Importantly, the NK cell population from infected lungs of *Ifnar1*^-/-^ animals contained significantly lower percentage of IFN-γ-producing cells than that from WT mice (Fig 4C). The populations of CD4 and CD8 T cells were also lower in lungs of infected *Ifnar1*^-/-^ mice compared to WT controls (Fig 4D and 4F, S4C Fig) but these cells did not produce IFN-γ regardless of the genotype (Fig 4E and 4G, S4C Fig), as revealed by comparison with PBS-treated controls. The lower numbers of CD4 and CD8 T cells in *Ifnar1*^-/-^ mice can be explained be impaired expression of the T cell chemokine *Cxcl10*. In sum, the absence of type I IFN signaling results in a defect in NK cell accumulation and NK cell-derived IFN-γ production in the lung of *K*. *pneumoniae*-infected mice.

### Alveolar macrophage priming by IFN-γ enhances killing of *K*. *pneumoniae*

**Fig 5:**
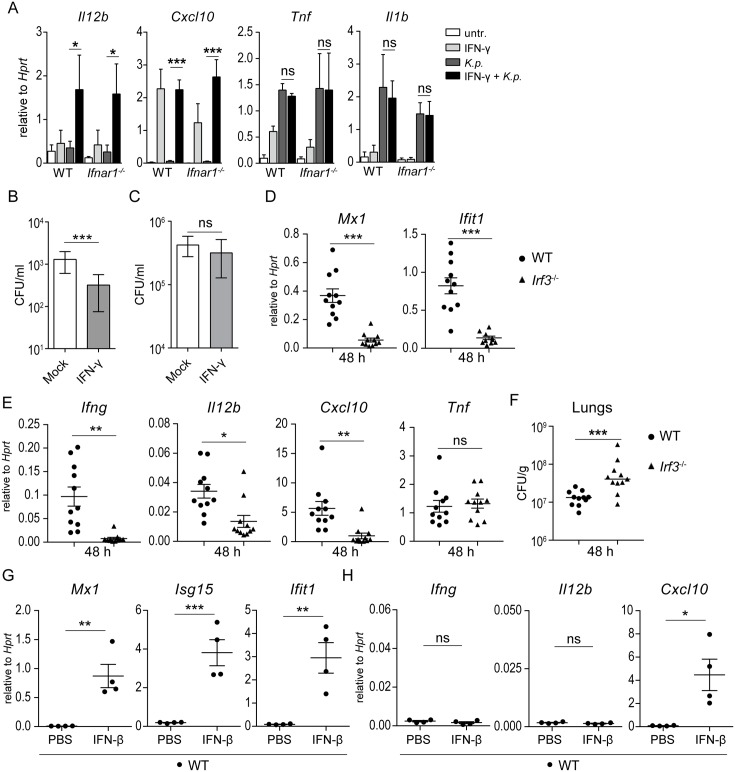
Priming by IFN-γ promotes macrophage activation against *K*. *pneumoniae*. **(A)** Alveolar macrophages from WT and *Ifnar1*^-/-^ mice were pretreated or not with 5 ng/ml mouse IFN-γ for 5 h, followed by infection (MOI = 70) for 1 h and RNA isolation. mRNA levels of *Il12b*, *Cxcl10*, *Tnf* and *Il1b* were quantitated by qPCR and normalized to *Hprt*. (**B,C**) Macrophages primed or unprimed with IFN-γ (10 ng/ml) for 2 h were infected (MOI = 70) for 90 min (including gentamicin treatment starting at 30 min after infection) (B) or 30 min without gentamicin treatment (C), and bacterial loads were determined as CFU per ml. **(D-F)** WT and *Irf3*^-/-^ mice were infected intranasally (5 x 10^4^ CFU of *K*. *pneumoniae*) for 48 h (n = 11 per genotype). Expression of indicated genes (D,E) and bacterial loads (F) in lungs were determined using qPCR and CFU assays, respectively. (**G, H**) WT mice received intranasally IFNβ (30 μl, 30,000 U) or PBS (n = 4 per treatment). Animals were euthanized 6 h following treatment. RNA was isolated from lungs and analyzed for expression of *Mx1*, *Isg15* and *Ifit1* (G) as well as *Ifng*, *Il12b* and *Cxcl10* (H). Statistical evaluation in (A), (D), (E), (G) and (H): unpaired Student’s *t* test; error bars, mean ± SEM; *, P < 0.05; **, P < 0.01; ***, P < 0.001; ns, not significant. Statistical evaluation in (B), (C) and (F): Mann-Whitney test; horizontal bars represent median; ***, P < 0.001; ns, not significant.

Macrophages require IFN-γ and/or IFN-γ priming for a complete anti-microbial response and for expression of the NK- and T cell-activating cytokine IL-12 and the NK and T cell chemoattractant CXCL10 [35, 36]. These responses were insufficiently activated in *Ifnar1*^*-/-*^ mice (Figs 3 and 4) suggesting that the impaired IFN-γ production in *Ifnar1*^*-/-*^ mice was causally involved in the low IL-12 and CXCL10 expression, and in the failure to control bacterial growth. To test this hypothesis, we isolated alveolar macrophages from WT and *Ifnar1*^*-/-*^ mice, primed them with IFN-γ and infected subsequently with *K*. *pneumoniae*. Both WT and *Ifnar1*^*-/-*^ alveolar macrophages primed for 5 h with IFN-γ induced *Il12b* upon *K*. *pneumoniae* infection (Fig 5A). Without priming, no *Il12b* was induced (Fig 5A). *Cxcl10* was induced by IFN-γ alone in both WT and *Ifnar1*^*-/-*^ alveolar macrophages (Fig 5A), consistent with the known direct activation of *Cxcl10* by IFN-γ [36]. In contrast, *Tnf* and *Il1b* were induced regardless of priming (Fig 5A). To assess the effect of priming on the anti-bacterial activity, IFN-γ–primed or mock-treated macrophages were infected, treated with gentamicin 1 h p.i. and incubated for additional 2 h. As anticipated, priming of macrophages with IFN-γ for 2 h resulted in a significant decrease in the number of intracellular bacteria at 3 h p.i. (Fig 5B) confirming the activating effect of IFN-γ on anti-bacterial macrophage activity [37]. Adhesion and uptake of bacteria were not affected by IFN-γ treatment (Fig 5C). Together, these results suggest that the key function of type I IFN production and signaling in defense against *K*. *pneumoniae* infection is the induction of IFN-γ. Since we found that the TLR4-IRF3 axis drives type I IFN production and, consequently, type I IFN signaling in *K*. *pneumoniae*-infected BMDMs (Fig 2) we asked whether IRF3 is required for IFN-γ induction and host defense in vivo. Infection of *Irf3*^*-/-*^ mice confirmed an impairment in *Mx1* and *Ifit1* but not *Tnf* induction (Fig 5D) suggesting that IRF3 is critically involved in *K*. *pneumoniae*-elicited type I IFN production in vivo. Moreover, *Irf3*^-/-^ mice exhibited impaired expression of *Ifng*, *Il12b* and *Cxcl10* and reduced ability to control bacterial growth (Fig 5E and 5F) demonstrating that deficiency in type I IFN induction has similar consequences for IFN-γ production as the lack of *Ifnar1*. Interestingly, however, direct triggering of type I IFN signaling by intranasally administered IFN-β into WT mice in the absence of *K*. *pneumoniae* did not activate *Ifng* gene expression in the lung despite a strong induction of ISGs as well as *Cxcl10* (Fig 5G). Thus, IFN-γ, which is required for efficient killing of *K*. *pneumoniae* by macrophages, is activated by type I IFN signaling only in the context of *K*. *pneumoniae* infection.

### Protective immune response against *K*. *pneumoniae* is independent of type I IFN signaling in alveolar macrophages and other myeloid cells

**Fig 6:**
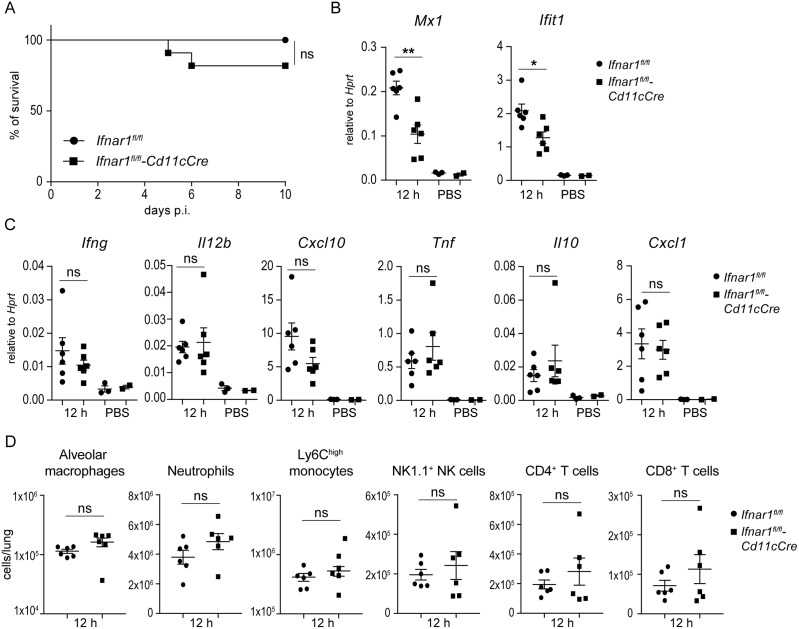
Protective responses against *K*. *pneumoniae* infection develop independently of type I IFN signaling in alveolar macrophages. (**A**) *Ifnar1*^*fl/fl*^-CD11cCre and *Ifnar1*^fl/fl^ mice (n = 11 and 10, respectively) were infected intranasally (5 x 10^4^ CFU of *K*. *pneumoniae*), and survival was monitored for 10 days. Kaplan-Meier survival curves are shown. Statistical evaluation: Log-rank (Mantel-Cox) test; ns, not significant. (**B, C**) *Ifnar1*^*fl/fl*^-CD11cCre and *Ifnar1*^fl/fl^ mice (n = 6 per genotype) were infected intranasally (5 x 10^4^ CFU of *K*. *pneumoniae*) or treated with PBS (n = 2 and 3, respectively) for 12 h. Expression of *Mx1* and *Ifit1* (B), and *Ifng*, *Il12b*, *Cxcl10*, *Tnf*, *Il10* and *Cxcl1* (B) in lungs was determined by qPCR (normalization to *Hprt*). Statistical evaluation: unpaired Student’s *t* test; error bars, mean ± SEM (n > 3); *, P < 0.05; **, P < 0.01; ns, not significant. (**D**) *Ifnar1*^*fl/fl*^-CD11cCre and *Ifnar1*^fl/fl^ mice (n = 6 per genotype) were infected intranasally (5 x 10^4^ CFU of *K*. *pneumoniae*) for 12 h or treated with PBS and immune cell subsets in lungs were analyzed by flow cytometry as in Fig 4. Populations are presented as total cells per lung calculated from percentages of individual immune cell subsets (shown in S5 Fig). Statistical evaluation: unpaired Student’s *t* test; error bars, mean ± SEM; ns, not significant.

Since alveolar macrophages produce and respond to type I IFNs during *K*. *pneumoniae* infection (Fig 2 and S2 Fig) we asked whether alveolar macrophage-intrinsic type I IFN signaling contributes to the immune response in vivo by using *Ifnar1*^*fl/fl*^-CD11cCre mice. *CD11c* promoter-driven of Cre recombinase expression results in the deletion of a loxP-flanked allele in conventional DCs (CD11c^high^) and alveolar macrophages [38, 39]. Consistently, *Ifnar1* was deleted in alveolar macrophages isolated from *Ifnar1*^*fl/fl*^-CD11cCre mice (S5A Fig). *Ifnar1*^*fl/fl*^-CD11cCre mice were similarly resistant against *K*. *pneumoniae* infection as *Ifnar1*^*fl/fl*^ controls (Fig 6B). Expression of the type I IFN target gene *Mx1* was significantly reduced in lungs of *Ifnar1*^*fl/fl*^-CD11cCre mice (Fig 6C) demonstrating that the CD11c^+^ cells represent a significant population of type I IFN-responding cells in the lung during *K*. *pneumoniae* infection. However, in agreement with the efficient defense, expression of *Ifng*, *Il12b*, and *Cxcl10* was not impaired in *Ifnar1*^*fl/fl*^-CD11cCre mice (Fig 6C). Expression of *Tnf*, *Il10* and *Cxcl1* was similar in both *Ifnar1*^*fl/fl*^-CD11cCre and *Ifnar*^fl/fl^ mice (Fig 6C). Flow cytometry analysis revealed comparable numbers of alveolar macrophages, neutrophils, inflammatory monocytes, NK cells as well as CD4 and CD8 T cells in lungs of *Ifnar1*^*fl/fl*^-CD11cCre and *Ifnar1*^fl/fl^ mice 12 h after *K*. *pneumoniae* infection (Fig 5D and S5B–S5D Fig). Furthermore, mice lacking *Ifnar1* in macrophages and neutrophils (*Ifnar1*^*fl/fl*^-LysMCre) or neutrophils only (*Ifnar1*^*fl/fl*^-MRP8Cre) exhibited similar clearance of *K*. *pneumoniae* as *Ifnar1*^*fl/fl*^ controls (S6A and S7A Figs). The expression of type I IFN responsive genes *Mx1* and *Ifit1* was reduced in *Ifnar1*^*fl/fl*^-LysMCre and to a lesser extent also in *Ifnar1*^*fl/fl*^-MRP8Cre mice (S6B and S7B Figs). Importantly, expression of *Ifng*, *Il12b*, and *Cxcl10* and the numbers of key immune cell subsets were not impaired in *Ifnar1*^*fl/fl*^-LysMCre and *Ifnar1*^*fl/fl*^-MRP8Cre mice (S6C–S6F and S6C–S6F Fig).

Together, type I IFN signaling in alveolar macrophages and in myeloid cells in general does not contribute to protective immune responses against *K*. *pneumoniae* although these cells generate a substantial part of the type I IFN signature in the infected lung.

### Type I IFN signaling promotes NK cell-mediated clearance of *K*. *pneumoniae*

**Fig 7:**
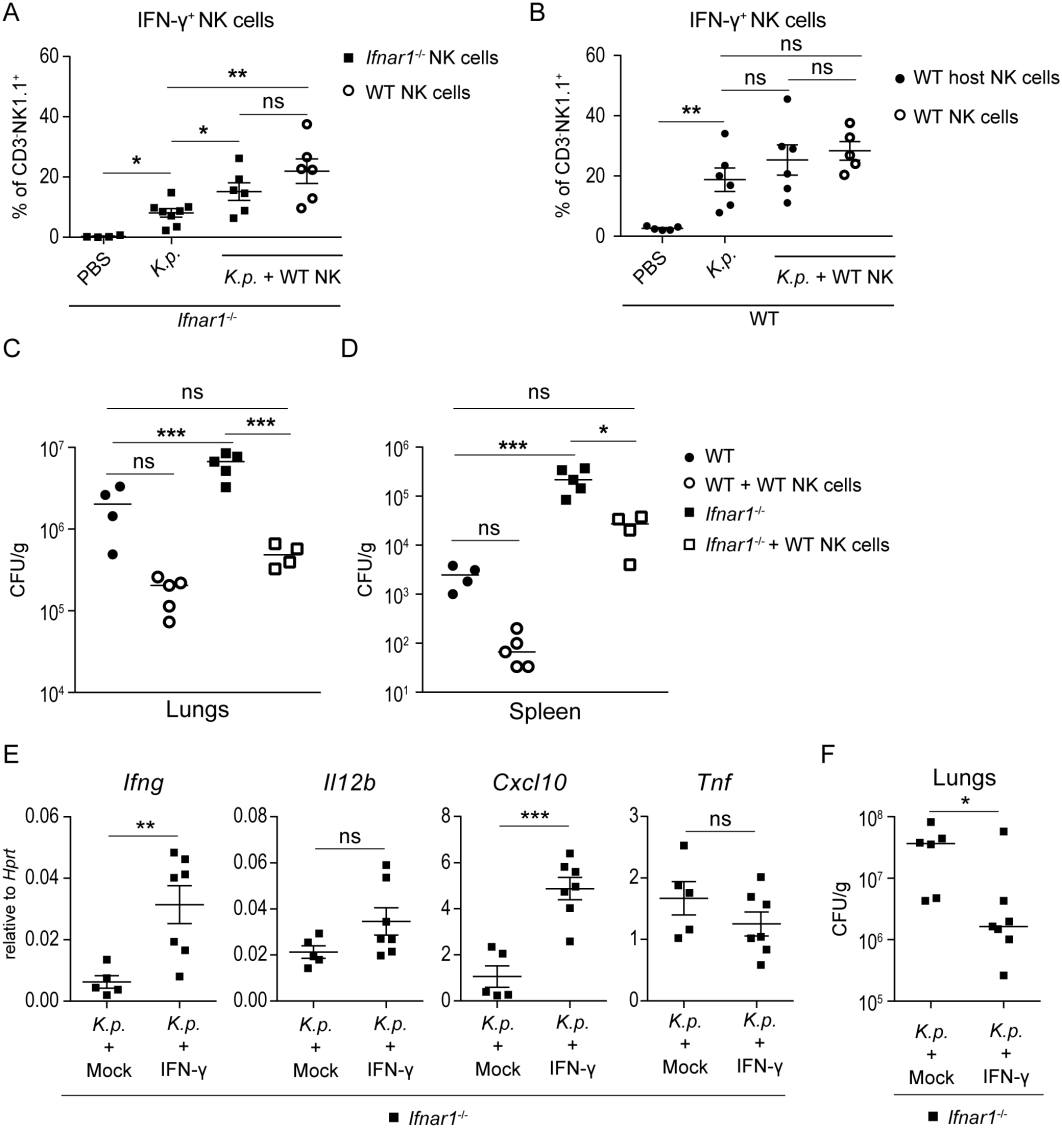
Transfer of WT NK cells or administration of IFN-γ restore control of *K*. *pneumoniae* growth in *Ifnar1*^*-/-*^ mice. (**A**) *Ifnar1*^-/-^ mice were treated with PBS, infected intranasally (5 x 10^4^ CFU of *K*. *pneumoniae*) (*K*.*p*.), or given 1 x 10^6^ WT NK cells and infected intranasally (5 x 10^4^ CFU of *K*. *pneumoniae*) (*K*.*p*. + WT NK). Lungs were analyzed 24 h p.i. by flow cytometry for IFN-γ-producing endogenous *Ifnar1*^-/-^ NK cells (dot plot groups 1–3) and exogenous WT NK cells (dot plot group 4) shown as percent of IFN-γ^+^ in CD3^-^NK1.1^+^ cells. Statistical evaluation: dots represent individual mice; error bars, mean ± SEM; unpaired Student’s *t* test; *, P < 0.05; **, P < 0.01; ns, not significant. (**B**) WT mice were treated with PBS, infected intranasally (5 x 10^4^ CFU of *K*. *pneumoniae*) (*K*.*p*.), or given 1 x 10^6^ WT NK cells and infected intranasally (5 x 10^4^ CFU of *K*. *pneumoniae*) (*K*.*p*. + WT NK). Lungs were analyzed 24 h p.i. by flow cytometry for IFN-γ-producing endogenous WT NK cells (dot plot groups 1–3) and exogenous WT NK cells (dot plot group 4) shown as percent of IFN-γ^+^ in CD3^-^NK1.1^+^ cells. Statistical evaluation: dots represent individual mice; unpaired Student’s *t* test; **, P < 0.01; ns, not significant. (**C, D**) Bacterial loads in lungs (C) and spleen (D) of infected WT and *Ifnar1*^-/-^ mice which received WT NK cells or were mock-treated. Data show CFU per g of lung per infected animal. Dots in dot plots represent individual mice. Statistical evaluation: one-way ANOVA with multiple comparisons; *, P < 0.05; ***, P < 0.001; ns, not significant. (**E, F**) *Ifnar1*^-/-^ mice infected intranasally (5 x 10^4^ CFU of *K*. *pneumoniae*) and given IFN-γ or PBS (mock) at the time of infection. Mice were euthanized 24 h p.i. and mRNA expression (*Ifng*, *Il12*, *Cxcl10*, *Tnf*) (E) and bacterial loads (F) in lungs were determined by qPCR and CFU assays, respectively. Statistical evaluation in (E) (n = 5, mock; n = 7, IFN-γ): unpaired Student’s *t* test; error bars, mean ± SEM; **, P < 0.01; ***, P < 0.001; ns, not significant. Statistical evaluation (F) (n = 6, mock; n = 7, IFN-γ): Mann-Whitney test; *, P < 0.05.

To find out whether the impaired activation and accumulation of NK cells in *Ifnar1*^*-/-*^ animals were causatively involved in the increased susceptibility to *K*. *pneumoniae* infection, we carried out NK cell transfer experiments. NK cells isolated from WT mice were adoptively transferred into *Ifnar1*^*-/-*^ mice using 1 x 10^6^ NK cells per recipient animal. Following *K*. *pneumoniae* infection, lungs of recipient mice were examined for NK cell accumulation, IFN-γ production by NK cells and bacterial burden. The numbers of donor WT NK cells in lungs of recipient *Ifnar1*^*-/-*^ mice were higher than the numbers of recipients’ own NK cells (i.e., *Ifnar1*^*-/-*^ NK cells) (S8 Fig), consistent with the observation that NK cell accumulation was higher in WT mice than in *Ifnar1*^*-/-*^ animals (Fig 4B). Importantly, the recipient *Ifnar1*^*-/-*^ mice showed similar percentages of IFN-γ-producing exogenous WT and endogenous *Ifnar1*^*-/-*^ NK cells, and both of these percentages were significantly higher than the percentage of IFN-γ-producing NK cells in *Ifnar1*^*-/-*^ mice without adoptive transfer (Fig 7A). Thus, the transfer of WT NK cells raised the percentage of IFN-γ-producing endogenous *Ifnar1*^*-/-*^ NK cells. Finally, *Ifnar1*^*-/-*^ mice that received WT NK cells exhibited approximately 10 times lower bacterial loads in lungs and almost lacked dissemination to the spleen when compared to mice which did not receive WT NK cells (Fig 7C and 7D). WT NK cells transferred into WT animals exhibited substantial IFN-γ production (Fig 7B) but they did not significantly raise bacterial clearance (Fig 7C and 7D), which is in agreement with the efficient defense of WT mice against *K*. *pneumoniae* infection.

These results suggested that the diminished NK cell-derived IFN-γ production was causative of the impaired bacterial clearance in *Ifnar1*^*-/-*^ mice. To test this, *Ifnar1*^*-/-*^ mice were intranasally administered IFN-γ at the time of infection. The IFN-γ treatment boosted the expression of *Ifng* and *Cxcl10* (Fig 7E) and reduced bacterial loads in lungs (Fig 7F).

In sum, the data show that donor WT NK cells display similar properties in recipient *Ifnar1*^*-/-*^ mice as in WT mice. Thus, the deficient NK cell accumulation and lower percentage of IFN-γ-producing NK cells observed in *Ifnar1*^*-/-*^ mice (Fig 4B and 4C) result from a cell-autonomous defect. Moreover, *Ifnar1*-deficient NK cells regain the ability to produce IFN-γ if macrophage priming is accomplished by exogenous IFN-γ or by IFN-γ derived from transferred WT NK cells.

## Discussion

In this study, we examined the role and mechanisms of action of type I IFN signaling in the context of lung infection with *K*. *pneumoniae*, a pathogen with one of the highest emergence of antibiotic resistant strains [2–4]. Our findings identify type I IFN signaling as a key driver of the mutually activating crosstalk between NK cells and alveolar macrophages which ultimately results in bacterial clearance and successful host defense. This discovery reveals a previously unrecognized mechanism of type I IFN-mediated anti-bacterial immunity and sheds light into regulation of immune responses against an utmost challenging human pathogen.

The protective effect of type I IFNs against *K*. *pneumoniae*-triggered pneumonia is caused by different mechanisms than those reported for other bacterial pathogens with the same tropism. Defense against *S*. *pneumoniae*, a gram-positive pathogen, is dependent on type I IFN signaling in alveolar epithelial type II cells. These cells require type I IFN signals to resist the destructive and death-promoting environment elicited in the course of *S*. *pneumoniae* infection [12]. The absence of type I IFN signaling causes an excessive destruction of the lung epithelial barrier and subsequent massive systemic dissemination of the pathogen. The lung barrier function is supported by type I IFNs by their protective effects on epithelial tight junctions and by inhibition of bacterial transmigration [11]. The protective effects of type I IFNs against infection with the gram negative intracellular pathogen *L*. *pneumophila* result from inhibition of the intracellular replication of the pathogen in infected macrophages [15]. The key type I IFN effector in this context appears to be the bactericidal itaconic acid which is produced by an enzyme encoded by the type I IFN target gene *Irg1* [15]. In contrast, the protective effects of type I IFNs against *K*. *pneumoniae* described in our study result from NK cell-dependent IFN-γ-mediated restriction of bacterial growth. IFN-γ controls *K*. *pneumoniae* growth in the lung but the precise mechanism has not been elucidated [22]. Lung failure resulting from exacerbated infection-elicited tissue destruction is a critical aspect of pneumonia since mitigation of lung injury and/or lessening of inflammation is associated with disease amelioration [40, 41]. Thus, the higher and progressively increasing bacterial burden and tissue injury in the lung of *K*. *pneumoniae*-infected Ifnar1-deficient mice suggest that these mice ultimately suffer respiratory failure.

It is well established that type I IFNs are typically induced following intracellular sensing of invading and/or phagocytosed bacteria thereby resembling induction by viruses, i.e. obligatory intracellular pathogens [9, 10]. This intracellular signaling principle applies also to the cell wall component LPS which activates the IFN-β-inducing TBK1-IRF3 pathway upon signaling emanating from the LPS-TLR4 complex localized in the endosomal membrane [42]. Our study reveals that this intracellular signaling is also the driver of type I IFN induction by *K*. *pneumoniae* which triggers the TBK/IRF3 pathway upon TLR4-dependent recognition of LPS and the capsule polysaccharide (CPS). Most common bacterial inducers of type IFNs are cell wall components (e.g. LPS) and nucleic acids (both RNA and DNA) [10]. The capacity of individual inducers to activate type I IFN production appears to be different in different innate immune cell types [13] leaving open the possibility that *K*. *pneumoniae* employs other inducers in addition to LPS and CPS in vivo. Interestingly, the *K*. *pneumoniae*-induced type I IFNs have no autocrine functions since mice lacking Ifnar1 in alveolar macrophages, the key sentinel cells in the lung [43], are similarly resistant to infection as Ifnar1-proficient mice. This is in marked contrast to skin infection with *S*. *pyogenes* in which myeloid cells are both the key type I IFN producer and effector cells [14]. Our data demonstrate that type I IFNs promote production of IFN-γ, a critical activator of antimicrobial effector functions of macrophages, by NK cells in *K*. *pneumoniae* infection. The mechanism of antimicrobial macrophage activation by IFN-γ involves primarily the stimulation of oxidative burst but other effector functions are emerging [37, 44]. For example, the IFN target genes GBPs (guanylate-binding proteins), which are linked to phagosomal processes, and IFN-γ-stimulated metabolic reprogramming have been implicated in microbial killing by macrophages [44, 45]. Interestingly, recent evidence demonstrates the ability of *K*. *pneumoniae* to manipulate phagosome maturation and survive antimicrobial attacks by macrophages [46] suggesting that IFN-γ might counteract such phagosome evasion mechanisms of *K*. *pneumoniae*. Future studies should investigate molecular mechanisms of *K*. *pneumoniae* eradication by macrophages in detail. Importantly, our data show that such mechanisms are activated by IFN-γ but not type I IFNs since autocrine type I IFN signaling in alveolar macrophages is dispensable for both macrophage priming and protective immune response. Type I IFN and type II IFN (i.e. IFN-γ) signaling pathways share the transcription factor STAT1, raising the therapeutically important question of STAT1 target genes acting as specific effectors of IFN-γ signaling during host defense against *K*. *pneumoniae* infection.

We provide complementary evidence for the fundamental importance of NK cell-autonomous type I IFN signaling in defense against *K*. *pneumoniae* infection. First, the use of *Ifnar1*^*fl/fl*^-Cd11cCre, *Ifnar1*^*fl/fl*^-LysMCre and *Ifnar1*^*fl/fl*^-MRP8Cre mouse strains allows the conclusion that type I IFN signaling in none of the major myeloid cell subsets present in infected lungs contributes to IFN-γ and IL-12 production, and to host defense. Second, WT NK cells introduced into *Ifnar1*^*-/-*^ mice produce IFN-γ in the Ifnar1-deficient environment and promote bacterial clearance. The NK cell-intrinsic requirement for type I IFN signaling in IFN-γ production in the course of *K*. *pneumoniae* infection is unprecedented in the context of bacterial infections studied to date. Interestingly, *Ifnar1*^*-/-*^ NK cells retain the ability to induce IFN-γ in *K*. *pneumoniae*-infected *Ifnar1*^*-/-*^ mice, as evident from IFN-γ production by *Ifnar1*^*-/-*^ NK cells after adoptive transfer of WT NK cells. Classically, NK cell production of IFN-γ is triggered by IL-12 originating from various subsets of myeloid cells [47]. This implies that in context of the NK cell transfer experiment, the WT NK-derived IFN-γ restores macrophage priming and IL-12 production which in turn activate NK cells regardless of their type I IFN signaling capacity.

Type I IFNs can directly stimulate NK cells to produce IFN-γ in response to certain viral infections [48, 49]. It is speculated that this mode of IFN-γ production is relevant during infection with viruses which induce little or no IL-12 such as the lymphocytic choriomeningitis virus [48]. Type I IFN-mediated NK cell stimulation involves direct activation of the IFN-γ gene driver STAT4 by the type I IFN receptor-associated JAK kinases [50, 51]. Such activation has so far not been reported for bacterial infections. Our experiment showing that administration of IFN-β alone does not result in IFN-γ induction indicates that type I IFNs act on NK cells in concert with accessory signals generated during *K*. *pneumoniae* infection. This mechanism would resemble IL-12 production by myeloid cells which requires signals derived from the pathogen recognition and from IFN-γ. NK cells express several pattern recognition receptors, e.g. TLR2 and TLR4, which potentially provide means for activation by bacterial products [52, 53]. However, the relevance of TLRs for NK cell activation during bacterial infections is unclear as NK cell-autonomous MyD88-dependent TLR signaling does not contribute to NK cell stimulation [54]. Of emerging interest are NK cytotoxic receptors since the activating receptor NKp46 and the mouse ortholog NCR1 have recently been demonstrated to act as pattern recognition receptors for *Fusobacterium nucleatum* and *Candida glabrata* [55, 56]. Thus, activating NK cell receptors might provide accessory signals for induction of IFN-γ by type I IFNs in the course of bacterial diseases such as the *K*. *pneumoniae* lung infection described in this study. The prominent role of type I IFN signaling in IFN-γ induction is in line with increased susceptibility of TRIF-deficient mice to *K*. *pneumoniae* infection [57]. TRIF-deficient mice exhibit impaired IFN-γ induction during *K*. *pneumoniae* infection and display improved resistance if treated with exogenous IFN-γ [57]. Our data showing the requirement for TRIF in IFN-β induction suggest that the impairment of IFN-γ production in TRIF-deleted mice is secondary to the deficient type I IFN (e.g. IFN-β) production in these mice. The exceptionally tight link between type I IFN signaling and NK cell IFN-γ production might represent a specific feature of defense against *K*. *pneumoniae*.

By virtue of their inhibition of viral replication type I IFNs became the first cytokines to be used in therapy of human diseases, most notably in infections caused by the hepatitis C virus [58, 59]. In contrast, the use of type I IFNs or their effectors for therapy of infectious diseases caused by bacteria is currently precluded by their incompletely understood and disparate effects during bacterial infections. Our study reveals an unexpected dependence of NK cell IFN-γ production on NK cell-intrinsic type I IFN signaling during bacterial pneumonia. This mechanism of IFN-γ induction and the resulting anti-bacterial activation of macrophages are indispensable for successful defense against *K*. *pneumoniae* lung infection. The increasing isolation of multidrug-resistant *K*. *pneumoniae* strains makes an urgent priority to develop effective therapeutics based on new targets and concepts. *K*. *pneumoniae* is exemplary of the mismatch between unmet medical needs and the current antimicrobial research and development pipeline. Arguably, therapies targeting the immune responses which thereby circumvent antibiotic resistance are highly desirable. Our study reveals an important and therapeutically exploitable aspect of immune defense against *K*. *pneumoniae*. Importantly, clinical records reporting frequent *K*. *pneumoniae* infections in patients deficient in IL-12 [60] suggest that the type I IFN-driven communication network between IFN-γ-producing NK cells and IL-12-producing myeloid cells is relevant also for humans.

## Materials and methods

### Ethics statement

Animal experiments were carried out at the Queen’s University Belfast and Max F. Perutz Laboratories of the University of Vienna. Experiments involving mice at Queen’s University Belfast were approved by the Queen’s University Belfast’s Ethics Committee and conducted in accordance with regulations described in the UK government Animals Act 1986 under the Project License PPL2700 issued by the UK Home Office. Animals were randomized for interventions but researches processing the samples and analyzing the data were aware which intervention group corresponded to which cohort of animals. Experiments involving mice at the Max F. Perutz Laboratories of the University of Vienna were discussed with the institutional ethics committee and performed in accordance with the Austrian law for animal experiments (BGBl. I Nr. 114/2012) under the permissions BMWF-66.006/0006-II/3b/2013 and BMWFW-66.006/0019-WF/V/3b/2016 issued by the Austrian Ministry of Science to PK.

### Mice

Mice were bred and kept under specific pathogen free (SPF) conditions according to recommendations of the Federation of European Laboratory Animal Science Association (FELASA). *Ifnar1*^-/-^ mice have been previously described [61]. *Ifnar1*^fl/fl^-CD11c-Cre, *Ifnar1*^fl/fl^-LysMCre and *Ifnar1*^fl/fl^-MRP8Cre mice were obtained by crossing *Ifnar1*^*fl/fl*^ mice [62] with CD11c-Cre mice, LysMCre and MRP8Cre [38, 63, 64], respectively, and littermate Cre^+^ and Cre^-^ control mice were used. All mice were on C57BL/6 background. C57BL/6N wild type (WT) mice were purchased from Charles River (Vienna) and Harlan (Belfast). Experiments were carried out using 7–12 weeks old mice with age and gender being matched between genotypes. For experiments requiring anesthesia, a solution of 10 mg/ml ketamine and 1 mg/ml xylazine (aniMedica) in isotonic saline (Sigma) was injected intraperitoneally (i.p.).

### Bacterial strains and growth conditions

*K*. *pneumoniae* 52.145 is a clinical isolate (serotype O1:K2) belonging to the CC65K2 virulent clonal group [5, 65]. The isogenic capsule mutant, strain 52145-Δ*wca*_K2_, has been described [66]. The LPS O-polysaccharide mutants, targeting the *glf* glycolstransfrase essential for the LPS O-polysaccharide biosynthesis [67] were constructed by insertion mutagenesis using the *pir* replication dependent plasmid pSF100 (to be described elsewhere). Bacteria were grown in LB medium at 37°C and carbenicillin 50 μg/ml was added to the growth medium to grow O-polysaccharide mutants.

### Cell culture and cell infections

Immortalized BMDM (iBMDM) cells (BEI Resources, NIAID, NIH, wild-type, NR-9456; *Trif*^-/-^, NR-9566; *Tram*^-/-^, NR-9567; *Myd88*^*-/-*^, NR-15633; and *Trif*^*-/-*^*Tram*^*-/-*^ mice, NR-9568) were grown in Dulbecco’s Modified Eagle Medium (DMEM; Gibco 41965) supplemented with 10% heat-inactivated fetal calf serum (FCS), 100 U/ml penicillin, and 0.1 mg/ml streptomycin (Gibco) at 37°C in a humidified 5% CO_2_ incubator. Murine alveolar macrophages MH-S (ATCC, CRL-2019) were grown in RPMI 1640 tissue culture medium supplemented with 10% heat-inactivated fetal calf serum (FCS), 100 U/ml penicillin, and 0.1 mg/ml streptomycin (Gibco) and 10 mM HEPES (Sigma-Aldrich). Cells were routinely tested for *Mycoplasma* contamination. To isolate BMDMs, tibias and femurs from wild-type, *irf3*^-/-^ and *tlr4*^-/-^ knock-out mice were removed using a sterile technique and the bone marrow was flushed with fresh medium. To obtain macrophages, cells were plated in L929-conditioned medium and cultivated for 4–6 days. Medium was replaced with fresh supplemented media every 3 days. For infections, bacteria were adjusted to an OD_600_ of 1.0 in PBS and infections were performed using a multiplicity of infection (MOI) of 70 bacteria per cell. To synchronize infection, plates were centrifuged at 200 x g for 5 min. For incubation times longer than 60 min, bacteria were killed by addition of gentamicin (100 μg/ml) which was not removed until the end of the experiment.

### Alveolar macrophage isolation and stimulations

To isolate alveolar macrophages (AMs), mice were euthanized by anesthetic overdose, trachea was surgically exposed and cannulated (BD Angiocath) followed by flushing of lungs with 5 x 1 ml of cold PBS + 0.5 mM EDTA. Obtained bronchoalveolar lavage fluids (BALF) from mice of the same genotype (n ≥ 6) were pooled on ice. Cells were collected from BALF by centrifugation (10 min, room temperature, 400 x g), resuspended and cultured in RPMI + 10% FCS + 1% penicillin and streptomycin. After 2 h, adherent cells were > 95% AMs as determined by trypan blue staining and were seeded for stimulation at the final concentration of 2 x 10^5^ cells/ml in RPMI + 10% FCS. For priming, cells were pretreated with 5 ng/ml mouse IFN-γ (eBioscience) for 5 h, followed by infection (MOI = 70) for 1 h. To synchronize infection, plates were centrifuged at 200 x g for 5 min. One hour after treatment with gentamicin (150 μg/ml) cells were lysed using Isol-RNA Lysis Reagent (5 Prime) and RNA was isolated.

### Model of lung infection

Bacteria in the stationary phase (overnight culture) were sub-cultured and grown at 37°C with agitation to reach mid log phase. Subsequently, bacteria were harvested by centrifugation (20 min, 2500 x g, 24°C), resuspended in 1x PBS and adjusted to 5 x 10^4^–1 x 10^5^ colony forming units (CFU) per 30 μl as determined by plating 10-fold serial dilutions on LB plates. Mice were anesthetized and 30 μl of bacterial suspension were inoculated intranasally. Infected animals were monitored every 4 to 8 hours and were euthanized when reaching behavioral and/or pathophysiological humane endpoints. Survival was monitored for 10 days. For experiments other than survival, animals were euthanized at indicated time points by either cervical dislocation or anesthetic overdose.

### Determination of bacterial loads

To determine bacterial loads, mice were euthanized at indicated time points. Lungs, spleens and livers were aseptically removed, weighted and placed in cold 1 x PBS. After mechanical homogenization, 10-fold dilution series were prepared from organ homogenates and plated on LB plates. The following day, colonies were counted and bacterial load was calculated as colony forming units (CFUs) per gram of tissue.

### Histology

Mice were euthanized by anesthetic overdose. To collect lungs for histology trachea was surgically exposed and cannulated (BD Angiocath). 4% paraformaldehyde (PFA) was injected through trachea to inflate lungs, followed by their aseptic dissection from the thoracic cage. Inflated lungs were fixed overnight in excessive volume of 4% PFA, dehydrated, embedded in paraffin and 3 μm thick sections were prepared. Hematoxylin and eosin (H&E) staining of the sections was performed according to the standard protocol. H&E stained slides were evaluated by a board certified pathologist using an Axioskop 2 MOT microscope (Carl Zeiss). For additional review and image acquisition, representative slides were scanned using a *Pannoramic* Scan II slide scanner (3D Histech). Digital images were acquired with the Pannoramic Slide Viewer software (3D Histech). Sections were examined for airway inflammation (inflammatory cell infiltration of the intrapulmonary airways, alveolar ducts and alveoli), neutrophilic infiltration and intralesional bacterial burden (qualitative/semi-quantitative extent of bacterial presence within the regions of inflammation). Standard pathology criteria were used to score histomorphologic features of the lesion—degree of the lesion and the extent of involvement: score 0 –none/insignificant; score 1 –minimal; less than 10%, score 2 –mild; 10 to 30%, score 3 –moderate; 30–60% and score 4 –severe, more than 60%.

### Enzyme-linked immunosorbent assay (ELISA) and cytokine measurement

Lung homogenates of infected and uninfected mice were prepared as for determination of bacterial load in 1x PBS containing protease inhibitors (Complete protease inhibitor, Roche) and frozen at -80°C. Homogenates were subjected to two cycles of thawing and freezing, centrifuged (5 min, 16000 x g, 4°C) and supernatants were collected for further measurements. Cytokine concentrations of TNF, IL-1β, IFN-γ and IL-12p70 were measured in supernatants using DuoSet ELISA kits (R&D Systems). Kits were used according to the manufacturer’s instructions. Total protein in lungs was determined by Pierce BCA Protein Assay Kit (Thermo Scientific) and used for normalization purposes.

### Immunoblot analysis

Lysates were prepared in lysis buffer (1x SDS Sample Buffer, 62.5 mM Tris-HCl pH 6.8, 2% w/v SDS, 10% glycerol, 50 mM DTT, 0.01% w/v bromophenol blue). Proteins were resolved by standard 10% SDS-PAGE and electroblotted onto nitrocellulose membranes. Membranes were blocked with 4% bovine serum albumin (w/v) in TBST and protein bands were detected with specific antibodies using chemiluminescence reagents and a G:BOX Chemi XRQ chemiluminescence imager (Syngene). The following rabbit antibodies were used: anti-phospho IRF3 (Ser 396) (1:1000; Cell Signaling #4947), anti-phospho-TBK1 (Ser 172) (1:1000; Cell Signaling #5483), and anti-ISG15 (1:1000; Cell Signaling #9636). Immunoreactive bands were visualized by incubation with horseradish peroxidase-conjugated goat anti-rabbit immunoglobulins (1:5000) or goat anti-mouse immunoglobulins (1:1000; Bio-Rad). To ensure that equal amounts of proteins were loaded, blots were re-probed with α-tubulin (1:3000; Sigma-Aldrich). To detect multiple proteins, membranes were re-probed after stripping of previously used antibodies using a pH 2.2 glycine-HCl/SDS buffer.

### Type I IFN bioassay

Cells were seeded in (6-well plate; 1 x 10^6^ cells per well) and grown for 24 h. Cells were infected (MOI = 100) for the indicated time points, and supernatants were collected. Murine type I IFNs were detected using B16-Blue IFN-α/β cells (Invivogen) which carry a SEAP reporter gene under the control of the type I IFN-inducible ISG54 promoter enhanced by a multimeric ISRE. Levels of SEAP in the supernatants were determined as per the manufacturer’s instructions.

### Intracellular survival

*K*. *pneumoniae* intracellular survival was assessed as previously described with minor modifications [46]. Briefly, macrophages were seeded in 12-well tissue culture plates at a density of 2.5 x 10^5^ cells per well 15 h before the experiment. Bacteria were grown in 5-ml LB, harvested in the exponential phase (2500 x g, 20 min, 24°C), washed once with PBS and a suspension containing approximately 1 x 10^8^ CFU/ml was prepared in 10 mM PBS (pH 6.5). Cells were infected with 175 μl of this suspension to obtain MOI of 70 bacteria per cell in a final volume of 1 ml DMEM tissue culture medium supplemented with 10% heat-inactivated FCS and 10 mM Hepes. To synchronize infection, plates were centrifuged at 200 x g during 5 min. After 30 min of contact, cells were washed twice with PBS and incubated for additional 60 min with 1 ml tissue culture medium supplemented with gentamicin (100 μg/ml) to eliminate extracellular bacteria. Initial attachment of bacteria was assessed after 30 min contact as previously described [46]. To determine intracellular bacterial load, cells were washed three times with PBS and lysed with 300 μl of 0.05% saponin in PBS for 10 min at room temperature. Serial dilutions were plated on LB to quantify the number of intracellular bacteria. Intracellular bacterial load is represented as CFU per ml. All experiments were done on at least three independent occasions.

### RNA isolation, reverse transcription and quantitative PCR (qPCR)

Total RNA was isolated from lung homogenates and cells using Isol-RNA Lysis Reagent (5 Prime) or Trizol according to the manufacturer’s protocol. DNase digestion was performed using 10 U of recombinant DNase I (Roche). RNA was PolyA primed with Oligo (dT)_18_ primers (Eurofins Genomics) and reverse-transcribed using Mu-MLV reverse transcriptase (Fermentas). qPCRs were run on a Realplex Mastercycler (Eppendorf) or Stratagene Mx3005P qPCR System and cDNA was quantified by SYBR Green method using HOT FIREPol EvaGreen qPCR supermix (Medibena) or KAPA SYBR FAST qPCR Kit. mRNA expression of the housekeeping gene *Hprt* was used for normalization purposes. Primers for qPCR were (in 5’-3’ orientation):

*Hprt* fwd-GCAGTCCCAGCGTCGTGAT, rev-CAGGCAAGTCTTTCAGTCCTGTC

*Il12b* (p40) fwd-ACAGCACCAGCTTCTTCATCAG, rev-TCTTCAAAGGCTTCATCTGCAA

*Ifng* fwd-CGGCACAGTCATTGAAAGCC, rev-TGTCACCATCCTTTTGCCAGT

*Il1b* fwd-AGATGAAGGGCTGCTTCCAAA, rev-AATGGGAACGTCACACACCA

*Ifnb* fwd- TCAGAATGAGTGGTGGTTGC, rev- GACCTTTCAAATGCAGTAGATTCA

*Tnf* fwd-GATCGGTCCCCAAAGGGATG, rev-CACTTGGTGGTTTGCTACGAC

*Cxcl1* fwd-TGCACCCAAACCGAAGTCATAG, rev-TTGTATAGTGTTGTCAGAAGCCAGC

*Il10* fwd-GGACTTTAAGGGTTACTTGGGTTGCC, rev-CATGTATGCTTCTATGCAGTTGATGA

*Mx1* fwd-GACTACCACTGAGATGACCCAGC, rev- ATTTCCTCCCCAAATGTTTTCA

*Ifit1* fwd-CAGGTTTCTGAGGAGTTCTG, rev-TGAAGCAGATTCTCCATGAC

*Isg15* fwd-GGGGCCACAGCAACATCTAT, rev-CGCTGGGACACCTTCTTCTT

*Cxcl10* fwd-TGCGAGCCTATCCTGCCCACGTG, rev-CCGGGGTGTGTGCGTGGCTTCA

*Ifnar1* fl/fl fwd-GCCCTGCTGAATAAGACCAG, rev-ACTGGCCTCAAACTCACTGC

### Flow cytometry

To prepare whole lung cell suspensions, lungs were cut to pieces with scissors and digested in RPMI containing 10% FCS, 1 mg/ml collagenase I (Roche) and 0.25 mg/ml DNase I (Roche) for 1 h at 37°C with agitation. Single-cell suspensions were obtained by flushing the samples through 70 μm strainer. Red blood cells were lysed using hypotonic shock and washed twice with PBS. To exclude dead cells, samples were stained with FVD eFluor 506 (eBioscience), prior to Fc blocking with anti-CD16/CD32 (2.4G2, BD). Suspensions were stained for cell surface proteins and intracellular IFN-γ in appropriate combinations of following monoclonal antibodies conjugated to allophycocyanin-eFluor 780, allophycocyanin, brilliant violet 711, phycoerythrin, brilliant violet 421, phycoerythtrin-cyanine7, peridinin chlorophyll protein-cyanine 5.5 and fluorescein isothiocyanate: anti-CD45 (30-F11), anti-CD11c (HL3), anti-SiglecF (E50-2440), anti-Ly6G (1A8), anti-Ly6C (HK1.4), anti-CD11b (M1/70), anti-CD3 (11-26c(11–26)), anti-CD8 (53–6.7), anti-NK1.1 (PK136) and anti IFN-γ (XMG1.2), purchased from BD, eBioscience and Biolegend. To prepare cells for intracellular staining, suspensions were incubated in fixation/permeabilization buffer (eBioscience), followed by Fc blocking, washing and staining in permeabilization buffer (eBioscience). Dead cells were excluded based on their light-scattering characteristics and FVD staining. Cell doublets were excluded based on FSC-H/FSC-A and SSC-H/SSC-A. All data acquisitions were performed using LSR Fortessa II (BD) cytometer interfaced with FACSDiva. FlowJo X (Tree Star) software was used for data analysis and graphical representation.

### Sorting of alveolar macrophages

Mice were either infected or given PBS and euthanized 24 h post-treatment. Lungs were harvested, and dissociated using VDI 12 tissue homogeniser (VWR) in sterile PBS. Single-cell suspensions were obtained by flushing the samples through 70 μm strainer. To exclude dead cells, samples were stained with FVD eFluor 506 (eBioscience), prior to Fc blocking with anti-CD16/CD32 (2.4G2, BD). To sort out CD45^+^CD11c^high^SiglecF^+^ alveolar macrophages, cell suspensions were stained for cell surface proteins using monoclonal antibodies conjugated to allophycocyanin-eFluor 780, allophycocyanin and brilliant violet 421: anti-CD45 (30-F11), anti-CD11c (N418) and anti-SiglecF (E50-2440), purchased from BD Bioscience, eBioscience and Biolegend. RNA from sorted alveolar macrophages was extracted using Power SYBR Green Cells-to-CT Kit (4402954, Ambion) according to manufacturer’s instructions.

### NK isolation and adoptive transfer

Splenic NK cells from WT mice were isolated by magnetic bead labeling (CD49b^+^ DX5 microbeads) following manufacturer’s instructions (Miltenyi Biotec). Before magnetic separation, 1 x 10^7^ cells were labeled with CellTrace CFSE kit (Thermo) according to the manufacturer’s protocol. Labeled NK cells (1 x 10^6^ cells/mouse) were adoptively transferred intravenously into *ifnar1*^*-*/-^ mice, which were infected intranasally with 3 x 10^5^ *K*. *pneumoniae* 52.145 CFU. 24 h post infection mice were euthanized and bacterial loads in lungs determined. Intracellular IFN-γ was detected using anti IFN-γ (XMG1.2). Cells were analyzed using a FACSCantoII flow cytometer and FlowJo software (Tree Star).

### IFN-γ treatment of animals

For IFN-**γ** rescue analysis, *Ifnar1*^-/-^ mice were infected with standard inoculum, and immediately post-infection intranasally given 20 μl of PBS, or 100 ng rIFN-γ in 20 μl of PBS (recombinant Mouse IFN-gamma Protein, R&D, Carrier Free, cat. number 485-MI-100, LOT#CFP2516032). Mice were euthanized 24 h p.i. and bacterial loads and mRNA expression in lungs were determined.

### IFN-β treatment of animals

Mice were anesthetized and inoculated intranasally with 30 μl PBS containing 30,000 U carrier-free recombinant mouse IFN-β (PBL Interferon Source, LOT#6450). Control animals received 30 μl of PBS. 6 h post-treatment animals were euthanized and total RNA was isolated from the lungs.

### Statistics

Data analysis, statistical testing and visualization was performed with Prism 6 (GraphPad Software) using Log-rank Mantel-Cox test, Mann-Whitney test, unpaired two-tailed Student’s *t* test and One-way ANOVA with multiple comparisons, as indicted in the figure legends. Medians are depicted as horizontal bars, and means are depicted as horizontal bars ± SEM. Statistical significance is indicated as follows: ns (not significant), P > 0.05, *, P < 0.05; **, P < 0.01; ***, P < 0.001.

## Supporting information

S1 Fig (related to Fig 1): Histopathology showing lungs from PBS-treated WT and *Ifnar1*^-/-^ mice and from *Ifnar1*^-/-^ mice with different degree of *K*. *pneumoniae*–caused bronchopneumonia. **(A)** H&E-stained representative sections of lungs of WT and *Ifnar1*^-/-^ animals that were given intranasal PBS. Note no difference in lung histology between genotypes. Magnification: 3x; insets 80x. (**B**) H&E-stained representative sections of lungs of *Ifnar1*^-/-^ animals that (1) approached humane endpoints (left panel); (2) at the same time were showing mild symptoms (middle panel) and (3) survived longer than 10 days (right panel). Black arrows and B indicate aggregates of bacilli; white arrows and N indicate neutrophils. Magnification 1x, 20x and 40x; black bars are 5 mm, 50 μm and 20 μm, respectively.
(TIF)

S2 Fig (related to Fig 2). *K*. *pneumoniae*-induced type I IFN signaling including protein modification by ISG15 (ISGylation) is dependent on IFNAR1, TLR4, TRIF and TRAM. **(A)** Mouse alveolar macrophages (MH-S cell line) and BMDMs from WT mice were infected with *K*. *pneumoniae* (MOI = 70) for 1, 3 and 5 h, or left untreated, and type I IFN levels in the supernatant were determined. (n = 8). **(B)** BMDMs from WT and *Ifnar1*^-/-^ mice were infected for indicated time points with *K*. *pneumoniae*, or treated with IFN-γ (for positive control) or left untreated. ISG15 conjugates and tubulin (loading control) were detected by in whole cell extracts by Western blotting. **(C, D)** BMDMs from WT and *Tlr4*^-/-^ mice (C) or *Tram*^-/-^*Trif*^-/-^ mice (D) were infected for 12 h with *K*. *pneumoniae*, or left untreated. ISG15 conjugates, ISG15 protein and tubulin (loading control) were detected in whole cell extracts by Western blotting. **(E)** WT, *Tram*^-/-^ and *Trif*^-/-^ BMDMs were infected as in (A) for indicated time points, or left untreated. *Ifnb*, *Mx1*, *Ifit1* and *Isg15* mRNA levels were determined by qPCR and normalized to *Hprt*. **(F)** WT, *Tram*^-/-^, *Trif*^-/-^ and *Myd88*^-/-^ BMDMs were infected as in (A) for indicated time points, or left untreated. p-TBK1, p-IRF3 and tubulin (loading control) were detected in whole cell extracts by Western blotting. (**G**) BMDMs were infected as in (A) with a *cps*, O-polysaccharide, and double *cps*-O-polysaccharide *K*. *pneumoniae* mutants for indicated time points, or left untreated. p-TBK1, p-IRF3 and tubulin (loading control) were detected in whole cell extracts by Western blotting. Statistical evaluation in (E): unpaired Student’s *t* test; error bars, mean ± SEM (n > 3).
(TIF)

S3 Fig (related to Fig 3). IFN-γ and IL-12 cytokine production, and *Cxcl1* and *Il1b* expression in lungs of *K*. *pneumoniae*-infected WT and *Ifnar1*^-/-^ mice. WT and *Ifnar1*^-/-^ mice were infected intranasally (5 x 10^4^ CFU of *K*. *pneumoniae*) for 12 **(A, B)** or 48 (**C**) h, or treated with PBS. (A) IFN-γ, IL-12 (p70), TNF and IL-1β protein levels in lungs determined by ELISA. (B, C) *Cxcl1* and *Il1b* mRNA levels determined by qPCR (normalized to *Hprt*). Statistical evaluation: unpaired Student’s *t* test; error bars, mean ± SEM (n > 3); *, P < 0.05; **, P < 0.01; ***, P < 0.001; ns, not significant.
(TIF)

S4 Fig (related to Fig 4). Representative flow cytometry plots of alveolar macrophages, neutrophils, monocytes, CD4 T, CD8 T cells, IFN-γ^+^ CD4 and IFN-γ^+^ CD8 T cells. **(A, B)** Flow cytometry plots (representative experiments) and percentages of CD45^+^ cells for SiglecF^+^CD11c^high^ alveolar macrophages (A) and neutrophils (Cd11b^+^Ly6G^+^Ly6C^med^) with inflammatory monocytes (CD11b^+^Ly6G^-^Ly6C^high^) (B) are shown. **(C)** Representative flow cytometry plots of CD3^+^CD4^+^, CD3^+^CD8^+^, IFN-γ^+^ CD4 and IFN-γ^+^ CD8 T cells. Numbers indicate percentages in the outlined area of live CD45^+^ cells.
(TIF)

S5 Fig (related to Fig 6). Analysis of *Ifnar1*^fl/fl^-CD11cCre mice. (**A**) PCR of genomic DNA isolated from alveolar macrophages (AMs) and tails of *Ifnar1*^fl/fl^-CD11cCre mice *Ifnar1*^fl/fl^ mice. Indicated are 1000 and 3000 bp bands, as well as a deletion band (1300 bp). **(B-D)** *Ifnar1*^*fl/fl*^-CD11cCre and *Ifnar1*^fl/fl^ mice (n = 6 per genotype) were infected intranasally (5 x 10^4^ CFU of *K*. *pneumoniae*) for 12 h, and immune cell subsets in lungs were analyzed by flow cytometry. Representative flow cytometry plots of alveolar macrophages (SiglecF^+^CD11c^high^) (B, left panels), neutrophils (Cd11b^+^Ly6G^+^Ly6C^med^) and inflammatory monocytes (CD11b^+^Ly6G^-^Ly6C^high^) (C, left panels), NK cells (CD3^-^NK1.1^+^) (D, left panels), CD4 T cells (CD3^+^CD4^+^) and CD8 T cells (CD3^+^CD8^+^) (E, left panels) are shown. Numbers in the right panels indicate percentages of individual immune cell subsets in live CD45^+^ cells. Statistical evaluation: unpaired Student’s *t* test; error bars, mean ± SEM; ns, not significant.
(TIF)

S6 Fig (related to Fig 6). Analysis of *Ifnar1*^fl/fl^-LysMCre mice. (**A**) *Ifnar1*^*fl/fl*^-LysMCre and *Ifnar1*^fl/fl^ mice (n = 6 per genotype) were infected intranasally (5 x 10^4^ CFU of *K*. *pneumoniae*) for 48 h, and bacterial loads in lungs were determined. Statistical evaluation: Mann-Whitney test; ns, not significant. (**B, C**) *Ifnar1*^*fl/fl*^-LysMCre and *Ifnar1*^fl/fl^ mice (n = 5 and 6, respectively) were infected as in (A). RNA was isolated from lungs and analyzed for expression of *Mx1* and *Ifit1* (B) as well as *Ifng*, *Il12b*, *Cxcl10*, *Tnf*, *Il10* and *Cxcl1* (C). Statistical evaluation: unpaired Student’s *t* test; error bars, mean ± SEM; **, P < 0.01; ns, not significant. (**D-F**) *Ifnar1*^*fl/fl*^-LysMCre and *Ifnar1*^fl/fl^ mice (n = 5 per genotype) were infected as in (A). Immune cell subsets in lungs were analyzed by flow cytometry. Representative flow cytometry plots of alveolar macrophages (SiglecF^+^CD11c^high^), neutrophils (Cd11b^+^Ly6G^+^Ly6C^med^) and inflammatory monocytes (CD11b^+^Ly6G^-^Ly6C^high^) (D, left panels), NK cells (CD3^-^NK1.1^+^) (E, upper panel), CD4 T cells (CD3^+^CD4^+^) and CD8 T cells (CD3^+^CD8^+^) (F, upper panels) are shown. Numbers in the right panels (D) and lower panels (E, F) indicate total numbers of individual immune cell subsets in lungs calculated from percentages of live CD45^+^ cells. Statistical evaluation: unpaired Student’s *t* test; error bars, mean ± SEM; ns, not significant.
(TIF)

S7 Fig (related to Fig 6). Analysis of *Ifnar1*^fl/fl^-MRP8Cre mice. (**A**) *Ifnar1*^*fl/fl*^-MRP8Cre and *Ifnar1*^fl/fl^ mice (n = 6 and 4, respectively) were infected intranasally (5 x 10^4^ CFU of *K*. *pneumoniae*) for 48 h, and bacterial loads in lungs were determined. Statistical evaluation: Mann-Whitney test; ns, not significant. (**B, C**) *Ifnar1*^*fl/fl*^-MRP8Cre and *Ifnar1*^fl/fl^ mice (n = 6 and 4, respectively) were infected as in (A). RNA was isolated from lungs and analyzed for expression of *Mx1* and *Ifit1* (B) as well as *Ifng*, *Il12b*, *Cxcl10*, *Tnf*, *Il10* and *Cxcl1* (C). Statistical evaluation: unpaired Student’s *t* test; error bars, mean ± SEM; **, P < 0.01; ns, not significant. (**D-F**) *Ifnar1*^*fl/fl*^-MRP8Cre and *Ifnar1*^fl/fl^ mice (n = 6 and 4, respectively) were infected as in (A). Immune cell subsets in lungs were analyzed by flow cytometry. Representative flow cytometry plots of alveolar macrophages (SiglecF^+^CD11c^high^), neutrophils (Cd11b^+^Ly6G^+^Ly6C^med^) and inflammatory monocytes (CD11b^+^Ly6G^-^Ly6C^high^) (D, left panels), NK cells (CD3^-^NK1.1^+^) (E, upper panel), CD4 T cells (CD3^+^CD4^+^) and CD8 T cells (CD3^+^CD8^+^) (F, upper panels) are shown. Numbers in the right panels (D) and lower panels (E, F) indicate total numbers of individual immune cell subsets in lungs calculated from percentages of live CD45^+^ cells. Statistical evaluation: unpaired Student’s *t* test; error bars, mean ± SEM; ns, not significant.
(TIF)

S8 Fig WT NK cells numbers in *Ifnar1*^-/-^ recipient mice. *Ifnar1*^-/-^ mice were treated with PBS, infected intranasally (5 x 10^4^ CFU of *K*. *pneumoniae*) (*K*.*p*.), or given 1 x 10^6^ WT NK cells and infected intranasally (5 x 10^4^ CFU of *K*. *pneumoniae*) (*K*.*p*. + WT NK). Lungs were analyzed 24 h p.i. or treatment. Endogenous *Ifnar1*^-/-^ NK cells (dot plot groups 1–3), as well as exogenous WT NK cells (dot plot group 4) detected by flow cytometry as CD3^-^NK1.1^+^ cells are shown in percent of CD45^+^ cells. Statistical evaluation: unpaired Student’s *t* test; error bars, mean ± SEM; **, P < 0.01; ns, not significant.
(TIF)

## References

[1] Munoz-Price LS, Poirel L, Bonomo RA, Schwaber MJ, Daikos GL, Cormican M, et al Clinical epidemiology of the global expansion of Klebsiella pneumoniae carbapenemases. The Lancet infectious diseases. 2013;13(9):785–96. Epub 2013/08/24. doi: 10.1016/S1473-3099(13)70190-7 . 23969216 10.1016/S1473-3099(13)70190-7 PMC4673667

[2] Holt KE, Wertheim H, Zadoks RN, Baker S, Whitehouse CA, Dance D, et al Genomic analysis of diversity, population structure, virulence, and antimicrobial resistance in Klebsiella pneumoniae, an urgent threat to public health. Proc Natl Acad Sci U S A. 2015;112(27):E3574–81. Epub 2015/06/24. doi: 10.1073/pnas.1501049112 . 26100894 10.1073/pnas.1501049112 PMC4500264

[3] Chen L, Mathema B, Chavda KD, DeLeo FR, Bonomo RA, Kreiswirth BN. Carbapenemase-producing Klebsiella pneumoniae: molecular and genetic decoding. Trends in microbiology. 2014;22(12):686–96. Epub 2014/10/12. doi: 10.1016/j.tim.2014.09.003 . 25304194 10.1016/j.tim.2014.09.003 PMC4365952

[4] Wyres KL, Holt KE. Klebsiella pneumoniae Population Genomics and Antimicrobial-Resistant Clones. Trends in microbiology. 2016;24(12):944–56. Epub 2016/10/16. doi: 10.1016/j.tim.2016.09.007 . 27742466 10.1016/j.tim.2016.09.007

[5] Lery LM, Frangeul L, Tomas A, Passet V, Almeida AS, Bialek-Davenet S, et al Comparative analysis of Klebsiella pneumoniae genomes identifies a phospholipase D family protein as a novel virulence factor. BMC Biol. 2014;12:41 Epub 2014/06/03. doi: 10.1186/1741-7007-12-41 . 24885329 10.1186/1741-7007-12-41 PMC4068068

[6] Gonzalez-Navajas JM, Lee J, David M, Raz E. Immunomodulatory functions of type I interferons. Nature reviews. 2012;12(2):125–35. Epub 2012/01/10. doi: 10.1038/nri3133 . 22222875 10.1038/nri3133 PMC3727154

[7] McNab F, Mayer-Barber K, Sher A, Wack A, O'Garra A. Type I interferons in infectious disease. Nature reviews. 2015;15(2):87–103. Epub 2015/01/24. doi: 10.1038/nri3787 . 25614319 10.1038/nri3787 PMC7162685

[8] Kovarik P, Castiglia V, Ivin M, Ebner F. Type I Interferons in Bacterial Infections: A Balancing Act. Frontiers in immunology. 2016;7(652):652 Epub 2017/01/14. doi: 10.3389/fimmu.2016.00652 . 28082986 10.3389/fimmu.2016.00652 PMC5183637

[9] Monroe KM, McWhirter SM, Vance RE. Induction of type I interferons by bacteria. Cellular microbiology. 2010;12(7):881–90. Epub 2010/05/21. doi: 10.1111/j.1462-5822.2010.01478.x . 20482555 10.1111/j.1462-5822.2010.01478.x PMC2897911

[10] Boxx GM, Cheng G. The Roles of Type I Interferon in Bacterial Infection. Cell host & microbe. 2016;19(6):760–9. Epub 2016/06/10. doi: 10.1016/j.chom.2016.05.016 . 27281568 10.1016/j.chom.2016.05.016 PMC5847370

[11] LeMessurier KS, Hacker H, Chi L, Tuomanen E, Redecke V. Type I interferon protects against pneumococcal invasive disease by inhibiting bacterial transmigration across the lung. PLoS pathogens. 2013;9(11):e1003727 Epub 2013/11/19. doi: 10.1371/journal.ppat.1003727 . 24244159 10.1371/journal.ppat.1003727 PMC3820719

[12] Maier BB, Hladik A, Lakovits K, Korosec A, Martins R, Kral JB, et al Type I interferon promotes alveolar epithelial type II cell survival during pulmonary Streptococcus pneumoniae infection and sterile lung injury in mice. European journal of immunology. 2016;46(9):2175–86. Epub 2016/06/18. doi: 10.1002/eji.201546201 . 27312374 10.1002/eji.201546201 PMC5370074

[13] Gratz N, Hartweger H, Matt U, Kratochvill F, Janos M, Sigel S, et al Type I interferon production induced by Streptococcus pyogenes-derived nucleic acids is required for host protection. PLoS pathogens. 2011;7(5):e1001345 Epub 2011/06/01. doi: 10.1371/journal.ppat.1001345 . 21625574 10.1371/journal.ppat.1001345 PMC3098218

[14] Castiglia V, Piersigilli A, Ebner F, Janos M, Goldmann O, Dambock U, et al Type I Interferon Signaling Prevents IL-1beta-Driven Lethal Systemic Hyperinflammation during Invasive Bacterial Infection of Soft Tissue. Cell host & microbe. 2016;19(3):375–87. Epub 2016/03/11. doi: 10.1016/j.chom.2016.02.003 . 26962946 10.1016/j.chom.2016.02.003

[15] Naujoks J, Tabeling C, Dill BD, Hoffmann C, Brown AS, Kunze M, et al IFNs Modify the Proteome of Legionella-Containing Vacuoles and Restrict Infection Via IRG1-Derived Itaconic Acid. PLoS pathogens. 2016;12(2):e1005408 Epub 2016/02/02. doi: 10.1371/journal.ppat.1005408 . 26829557 10.1371/journal.ppat.1005408 PMC4734697

[16] Stanley SA, Johndrow JE, Manzanillo P, Cox JS. The Type I IFN response to infection with Mycobacterium tuberculosis requires ESX-1-mediated secretion and contributes to pathogenesis. J Immunol. 2007;178(5):3143–52. Epub 2007/02/22. . 17312162 10.4049/jimmunol.178.5.3143

[17] Manca C, Tsenova L, Freeman S, Barczak AK, Tovey M, Murray PJ, et al Hypervirulent M. tuberculosis W/Beijing strains upregulate type I IFNs and increase expression of negative regulators of the Jak-Stat pathway. J Interferon Cytokine Res. 2005;25(11):694–701. Epub 2005/12/02. doi: 10.1089/jir.2005.25.694 . 16318583 10.1089/jir.2005.25.694

[18] Mayer-Barber KD, Andrade BB, Barber DL, Hieny S, Feng CG, Caspar P, et al Innate and adaptive interferons suppress IL-1alpha and IL-1beta production by distinct pulmonary myeloid subsets during Mycobacterium tuberculosis infection. Immunity. 2011;35(6):1023–34. Epub 2011/12/27. doi: 10.1016/j.immuni.2011.12.002 . 22195750 10.1016/j.immuni.2011.12.002 PMC3246221

[19] Henry T, Brotcke A, Weiss DS, Thompson LJ, Monack DM. Type I interferon signaling is required for activation of the inflammasome during Francisella infection. J Exp Med. 2007;204(5):987–94. Epub 2007/04/25. doi: 10.1084/jem.20062665 . 17452523 10.1084/jem.20062665 PMC2118578

[20] Henry T, Kirimanjeswara GS, Ruby T, Jones JW, Peng K, Perret M, et al Type I IFN signaling constrains IL-17A/F secretion by gammadelta T cells during bacterial infections. J Immunol. 2010;184(7):3755–67. Epub 2010/02/24. doi: 10.4049/jimmunol.0902065 . 20176744 10.4049/jimmunol.0902065 PMC2879132

[21] Xiong H, Keith JW, Samilo DW, Carter RA, Leiner IM, Pamer EG. Innate Lymphocyte/Ly6C(hi) Monocyte Crosstalk Promotes Klebsiella Pneumoniae Clearance. Cell. 2016;165(3):679–89. Epub 2016/04/05. doi: 10.1016/j.cell.2016.03.017 . 27040495 10.1016/j.cell.2016.03.017 PMC4842125

[22] Moore TA, Perry ML, Getsoian AG, Newstead MW, Standiford TJ. Divergent role of gamma interferon in a murine model of pulmonary versus systemic Klebsiella pneumoniae infection. Infect Immun. 2002;70(11):6310–8. Epub 2002/10/16. doi: 10.1128/IAI.70.11.6310-6318.2002 . 12379710 10.1128/IAI.70.11.6310-6318.2002 PMC130357

[23] Happel KI, Dubin PJ, Zheng M, Ghilardi N, Lockhart C, Quinton LJ, et al Divergent roles of IL-23 and IL-12 in host defense against Klebsiella pneumoniae. J Exp Med. 2005;202(6):761–9. Epub 2005/09/15. doi: 10.1084/jem.20050193 . 16157683 10.1084/jem.20050193 PMC2212952

[24] Mizgerd JP, Skerrett SJ. Animal models of human pneumonia. Am J Physiol Lung Cell Mol Physiol. 2008;294(3):L387–98. Epub 2007/12/29. doi: 10.1152/ajplung.00330.2007 . 18162603 10.1152/ajplung.00330.2007

[25] Lawlor MS, Hsu J, Rick PD, Miller VL. Identification of Klebsiella pneumoniae virulence determinants using an intranasal infection model. Molecular microbiology. 2005;58(4):1054–73. Epub 2005/11/03. doi: 10.1111/j.1365-2958.2005.04918.x . 16262790 10.1111/j.1365-2958.2005.04918.x

[26] Takeuchi O, Akira S. Pattern recognition receptors and inflammation. Cell. 2010;140(6):805–20. Epub 2010/03/23. doi: 10.1016/j.cell.2010.01.022 . 20303872 10.1016/j.cell.2010.01.022

[27] Wieland CW, van Lieshout MH, Hoogendijk AJ, van der Poll T. Host defence during Klebsiella pneumonia relies on haematopoietic-expressed Toll-like receptors 4 and 2. The European respiratory journal. 2011;37(4):848–57. Epub 2010/07/24. doi: 10.1183/09031936.00076510 . 20650991 10.1183/09031936.00076510

[28] Bhan U, Ballinger MN, Zeng X, Newstead MJ, Cornicelli MD, Standiford TJ. Cooperative interactions between TLR4 and TLR9 regulate interleukin 23 and 17 production in a murine model of gram negative bacterial pneumonia. PLoS ONE. 2010;5(3):e9896 Epub 2010/04/03. doi: 10.1371/journal.pone.0009896 . 20360853 10.1371/journal.pone.0009896 PMC2845620

[29] Tomas A, Lery L, Regueiro V, Perez-Gutierrez C, Martinez V, Moranta D, et al Functional Genomic Screen Identifies Klebsiella pneumoniae Factors Implicated in Blocking Nuclear Factor kappaB (NF-kappaB) Signaling. J Biol Chem. 2015;290(27):16678–97. Epub 2015/05/15. doi: 10.1074/jbc.M114.621292 . 25971969 10.1074/jbc.M114.621292 PMC4505419

[30] March C, Moranta D, Regueiro V, Llobet E, Tomas A, Garmendia J, et al Klebsiella pneumoniae outer membrane protein A is required to prevent the activation of airway epithelial cells. J Biol Chem. 2011;286(12):9956–67. Epub 2011/02/01. doi: 10.1074/jbc.M110.181008 . 21278256 10.1074/jbc.M110.181008 PMC3060550

[31] Regueiro V, Moranta D, Campos MA, Margareto J, Garmendia J, Bengoechea JA. Klebsiella pneumoniae increases the levels of Toll-like receptors 2 and 4 in human airway epithelial cells. Infect Immun. 2009;77(2):714–24. Epub 2008/11/19. doi: 10.1128/IAI.00852-08 . 19015258 10.1128/IAI.00852-08 PMC2632040

[32] Yang FL, Yang YL, Liao PC, Chou JC, Tsai KC, Yang AS, et al Structure and immunological characterization of the capsular polysaccharide of a pyrogenic liver abscess caused by Klebsiella pneumoniae: activation of macrophages through Toll-like receptor 4. J Biol Chem. 2011;286(24):21041–51. Epub 2011/04/12. doi: 10.1074/jbc.M111.222091 . 21478151 10.1074/jbc.M111.222091 PMC3122165

[33] Zeng X, Moore TA, Newstead MW, Deng JC, Kunkel SL, Luster AD, et al Interferon-inducible protein 10, but not monokine induced by gamma interferon, promotes protective type 1 immunity in murine Klebsiella pneumoniae pneumonia. Infect Immun. 2005;73(12):8226–36. Epub 2005/11/22. doi: 10.1128/IAI.73.12.8226-8236.2005 . 16299319 10.1128/IAI.73.12.8226-8236.2005 PMC1307052

[34] Xu X, Weiss ID, Zhang HH, Singh SP, Wynn TA, Wilson MS, et al Conventional NK cells can produce IL-22 and promote host defense in Klebsiella pneumoniae pneumonia. J Immunol. 2014;192(4):1778–86. Epub 2014/01/21. doi: 10.4049/jimmunol.1300039 . 24442439 10.4049/jimmunol.1300039 PMC3995347

[35] Hayes MP, Wang J, Norcross MA. Regulation of interleukin-12 expression in human monocytes: selective priming by interferon-gamma of lipopolysaccharide-inducible p35 and p40 genes. Blood. 1995;86(2):646–50. Epub 1995/07/15. . 7605994

[36] Liu M, Guo S, Hibbert JM, Jain V, Singh N, Wilson NO, et al CXCL10/IP-10 in infectious diseases pathogenesis and potential therapeutic implications. Cytokine Growth Factor Rev. 2011;22(3):121–30. Epub 2011/08/02. doi: 10.1016/j.cytogfr.2011.06.001 . 21802343 10.1016/j.cytogfr.2011.06.001 PMC3203691

[37] Nathan CF, Murray HW, Wiebe ME, Rubin BY. Identification of interferon-gamma as the lymphokine that activates human macrophage oxidative metabolism and antimicrobial activity. J Exp Med. 1983;158(3):670–89. Epub 1983/09/01. 6411853. 6411853 10.1084/jem.158.3.670 PMC2187114

[38] Caton ML, Smith-Raska MR, Reizis B. Notch-RBP-J signaling controls the homeostasis of CD8- dendritic cells in the spleen. J Exp Med. 2007;204(7):1653–64. Epub 2007/06/27. doi: 10.1084/jem.20062648 . 17591855 10.1084/jem.20062648 PMC2118632

[39] Bennett CL, Clausen BE. DC ablation in mice: promises, pitfalls, and challenges. Trends in immunology. 2007;28(12):519–25. 17964853 10.1016/j.it.2007.08.011

[40] Jamieson AM, Pasman L, Yu S, Gamradt P, Homer RJ, Decker T, et al Role of tissue protection in lethal respiratory viral-bacterial coinfection. Science. 2013;340(6137):1230–4. Epub 2013/04/27. doi: 10.1126/science.1233632 . 23618765 10.1126/science.1233632 PMC3933032

[41] Gamradt P, Xu Y, Gratz N, Duncan K, Kobzik L, Hogler S, et al The Influence of Programmed Cell Death in Myeloid Cells on Host Resilience to Infection with Legionella pneumophila or Streptococcus pyogenes. PLoS pathogens. 2016;12(12):e1006032 Epub 2016/12/16. doi: 10.1371/journal.ppat.1006032 . 27973535 10.1371/journal.ppat.1006032 PMC5156374

[42] Kagan JC, Su T, Horng T, Chow A, Akira S, Medzhitov R. TRAM couples endocytosis of Toll-like receptor 4 to the induction of interferon-beta. Nat Immunol. 2008;9(4):361–8. Epub 2008/02/26. doi: 10.1038/ni1569 . 18297073 10.1038/ni1569 PMC4112825

[43] Quinton LJ, Mizgerd JP. Dynamics of lung defense in pneumonia: resistance, resilience, and remodeling. Annual review of physiology. 2015;77:407–30. Epub 2014/08/26. doi: 10.1146/annurev-physiol-021014-071937 . 25148693 10.1146/annurev-physiol-021014-071937 PMC4366440

[44] Kim BH, Chee JD, Bradfield CJ, Park ES, Kumar P, MacMicking JD. Interferon-induced guanylate-binding proteins in inflammasome activation and host defense. Nat Immunol. 2016;17(5):481–9. Epub 2016/04/20. doi: 10.1038/ni.3440 . 27092805 10.1038/ni.3440 PMC4961213

[45] Su X, Yu Y, Zhong Y, Giannopoulou EG, Hu X, Liu H, et al Interferon-gamma regulates cellular metabolism and mRNA translation to potentiate macrophage activation. Nat Immunol. 2015;16(8):838–49. Epub 2015/07/07. doi: 10.1038/ni.3205 . 26147685 10.1038/ni.3205 PMC4509841

[46] Cano V, March C, Insua JL, Aguilo N, Llobet E, Moranta D, et al Klebsiella pneumoniae survives within macrophages by avoiding delivery to lysosomes. Cellular microbiology. 2015;17(11):1537–60. Epub 2015/06/06. doi: 10.1111/cmi.12466 . 26045209 10.1111/cmi.12466

[47] Rivera A, Siracusa MC, Yap GS, Gause WC. Innate cell communication kick-starts pathogen-specific immunity. Nat Immunol. 2016;17(4):356–63. Epub 2016/03/24. doi: 10.1038/ni.3375 . 27002843 10.1038/ni.3375 PMC4949486

[48] Mack EA, Kallal LE, Demers DA, Biron CA. Type 1 interferon induction of natural killer cell gamma interferon production for defense during lymphocytic choriomeningitis virus infection. MBio. 2011;2(4). Epub 2011/08/11. doi: 10.1128/mBio.00169-11 . 21828218 10.1128/mBio.00169-11 PMC3150756

[49] Martinez J, Huang X, Yang Y. Direct action of type I IFN on NK cells is required for their activation in response to vaccinia viral infection in vivo. J Immunol. 2008;180(3):1592–7. Epub 2008/01/23. . 18209055 10.4049/jimmunol.180.3.1592

[50] Miyagi T, Gil MP, Wang X, Louten J, Chu WM, Biron CA. High basal STAT4 balanced by STAT1 induction to control type 1 interferon effects in natural killer cells. J Exp Med. 2007;204(10):2383–96. Epub 2007/09/12. doi: 10.1084/jem.20070401 . 17846149 10.1084/jem.20070401 PMC2118450

[51] Nguyen KB, Watford WT, Salomon R, Hofmann SR, Pien GC, Morinobu A, et al Critical role for STAT4 activation by type 1 interferons in the interferon-gamma response to viral infection. Science. 2002;297(5589):2063–6. Epub 2002/09/21. doi: 10.1126/science.1074900 . 12242445 10.1126/science.1074900

[52] Martinez J, Huang X, Yang Y. Direct TLR2 signaling is critical for NK cell activation and function in response to vaccinia viral infection. PLoS pathogens. 2010;6(3):e1000811 Epub 2010/03/20. doi: 10.1371/journal.ppat.1000811 . 20300608 10.1371/journal.ppat.1000811 PMC2837413

[53] Eriksson M, Meadows SK, Basu S, Mselle TF, Wira CR, Sentman CL. TLRs mediate IFN-gamma production by human uterine NK cells in endometrium. J Immunol. 2006;176(10):6219–24. Epub 2006/05/04. . 16670332 10.4049/jimmunol.176.10.6219

[54] Lucas M, Schachterle W, Oberle K, Aichele P, Diefenbach A. Dendritic cells prime natural killer cells by trans-presenting interleukin 15. Immunity. 2007;26(4):503–17. Epub 2007/04/03. doi: 10.1016/j.immuni.2007.03.006 . 17398124 10.1016/j.immuni.2007.03.006 PMC2084390

[55] Vitenshtein A, Charpak-Amikam Y, Yamin R, Bauman Y, Isaacson B, Stein N, et al NK Cell Recognition of Candida glabrata through Binding of NKp46 and NCR1 to Fungal Ligands Epa1, Epa6, and Epa7. Cell host & microbe. 2016;20(4):527–34. Epub 2016/10/14. doi: 10.1016/j.chom.2016.09.008 . 27736647 10.1016/j.chom.2016.09.008 PMC5492882

[56] Chaushu S, Wilensky A, Gur C, Shapira L, Elboim M, Halftek G, et al Direct recognition of Fusobacterium nucleatum by the NK cell natural cytotoxicity receptor NKp46 aggravates periodontal disease. PLoS pathogens. 2012;8(3):e1002601 Epub 2012/03/30. doi: 10.1371/journal.ppat.1002601 . 22457623 10.1371/journal.ppat.1002601 PMC3310798

[57] van Lieshout MH, Florquin S, Van't Veer C, de Vos AF, van der Poll T. TIR-Domain-Containing Adaptor-Inducing Interferon-beta (TRIF) Mediates Antibacterial Defense during Gram-Negative Pneumonia by Inducing Interferon-x03B3. J Innate Immun. 2015;7(6):637–46. Epub 2015/06/13. doi: 10.1159/000430913 . 26065469 10.1159/000430913 PMC6738753

[58] Isaacs A, Lindenmann J. Virus interference. I. The interferon. Proc R Soc Lond B Biol Sci. 1957;147(927):258–67. . 13465720 10.1098/rspb.1957.0048

[59] Katze MG, He Y, Gale M, Jr. Viruses and interferon: a fight for supremacy. Nature reviews. 2002;2(9):675–87. Epub 2002/09/05. doi: 10.1038/nri888 . 12209136 10.1038/nri888

[60] Pedraza S, Lezana JL, Samarina A, Aldana R, Herrera MT, Boisson-Dupuis S, et al Clinical disease caused by Klebsiella in 2 unrelated patients with interleukin 12 receptor beta1 deficiency. Pediatrics. 2010;126(4):e971–6. Epub 2010/09/22. doi: 10.1542/peds.2009-2504 . 20855390 10.1542/peds.2009-2504 PMC3005354

[61] Muller U, Steinhoff U, Reis LF, Hemmi S, Pavlovic J, Zinkernagel RM, et al Functional role of type I and type II interferons in antiviral defense. Science. 1994;264(5167):1918–21. Epub 1994/06/24. . 8009221 10.1126/science.8009221

[62] Kamphuis E, Junt T, Waibler Z, Forster R, Kalinke U. Type I interferons directly regulate lymphocyte recirculation and cause transient blood lymphopenia. Blood. 2006;108(10):3253–61. Epub 2006/07/27. doi: 10.1182/blood-2006-06-027599 . 16868248 10.1182/blood-2006-06-027599

[63] Passegue E, Wagner EF, Weissman IL. JunB deficiency leads to a myeloproliferative disorder arising from hematopoietic stem cells. Cell. 2004;119(3):431–43. Epub 2004/10/28. doi: 10.1016/j.cell.2004.10.010 . 15507213 10.1016/j.cell.2004.10.010

[64] Clausen BE, Burkhardt C, Reith W, Renkawitz R, Forster I. Conditional gene targeting in macrophages and granulocytes using LysMcre mice. Transgenic Res. 1999;8(4):265–77. Epub 2000/01/06. . 10621974 10.1023/a:1008942828960

[65] Brisse S, Fevre C, Passet V, Issenhuth-Jeanjean S, Tournebize R, Diancourt L, et al Virulent clones of Klebsiella pneumoniae: identification and evolutionary scenario based on genomic and phenotypic characterization. PLoS ONE. 2009;4(3):e4982 Epub 2009/03/26. doi: 10.1371/journal.pone.0004982 . 19319196 10.1371/journal.pone.0004982 PMC2656620

[66] Llobet E, Tomas JM, Bengoechea JA. Capsule polysaccharide is a bacterial decoy for antimicrobial peptides. Microbiology (Reading, England). 2008;154(Pt 12):3877–86. Epub 2008/12/03. doi: 10.1099/mic.0.2008/022301-0 . 19047754 10.1099/mic.0.2008/022301-0

[67] Koplin R, Brisson JR, Whitfield C. UDP-galactofuranose precursor required for formation of the lipopolysaccharide O antigen of Klebsiella pneumoniae serotype O1 is synthesized by the product of the rfbDKPO1 gene. J Biol Chem. 1997;272(7):4121–8. Epub 1997/02/14. . 9020123 10.1074/jbc.272.7.4121

