# Supplementary figures and images for: Natural killer cell-intrinsic type I IFN signaling controls Klebsiella pneumoniae growth during lung infection
Source: PLoS Pathog. 2017 Nov 7;13(11):e1006696. doi: 10.1371/journal.ppat.1006696 (PMC5675380; doi:10.1371/journal.ppat.1006696)

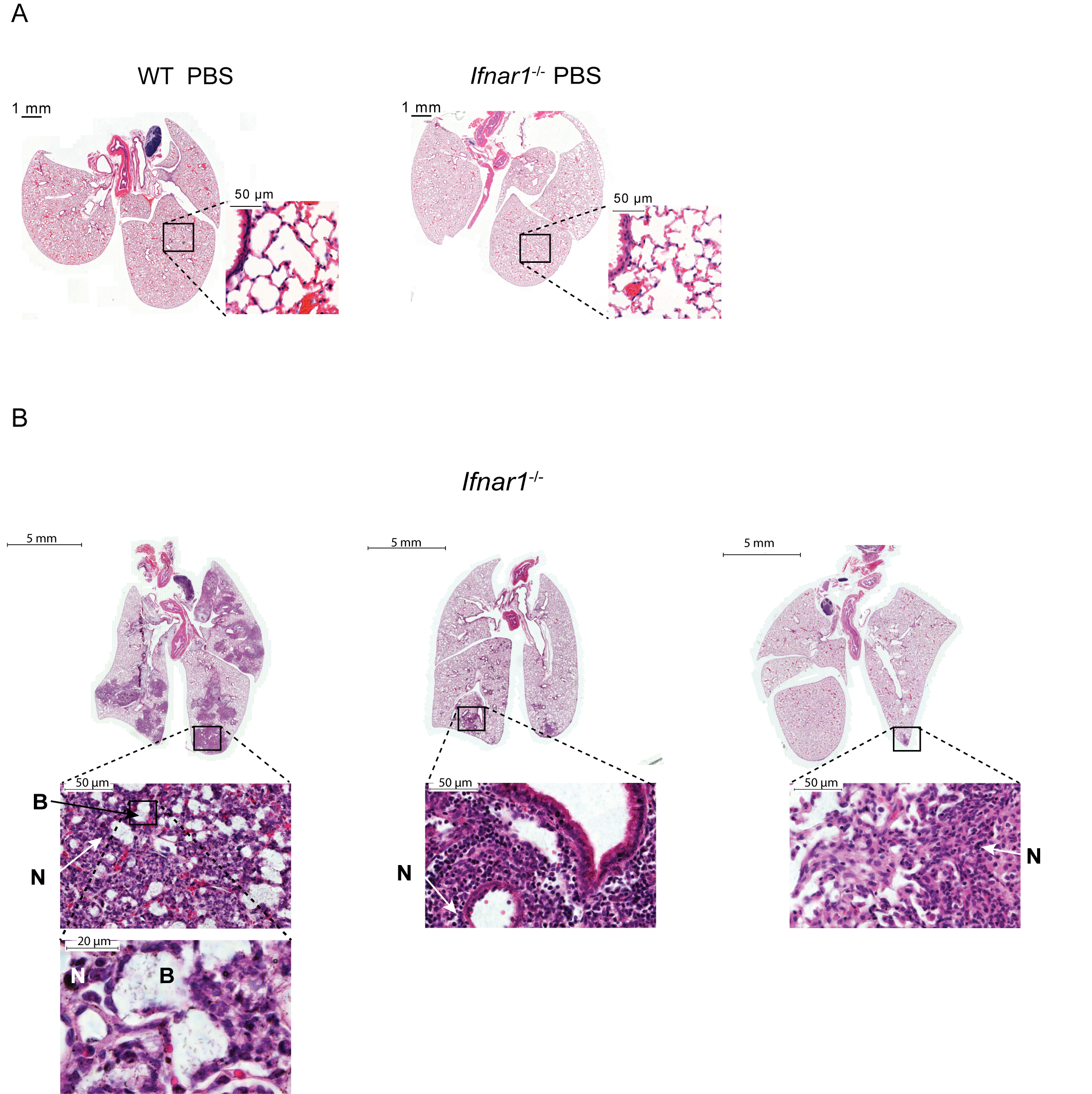

Supplement: S1 Fig — (A) H&E-stained representative sections of lungs of WT and Ifnar1-/- animals that were given intranasal PBS. Note no difference in lung histology between genotypes. Magnification: 3x; insets 80x. (B) H&E-stained representative sections of lungs of Ifnar1-/- animals that (1) approached humane endpoints (left panel); (2) at the same time were showing mild symptoms (middle panel) and (3) survived longer than 10 days (right panel). Black arrows and B indicate aggregates of bacilli; white arrows and N indicate neutrophils. Magnification 1x, 20x and 40x; black bars are 5 mm, 50 μm and 20 μm, respectively. (TIF) [file ppat.1006696.s001.tif]

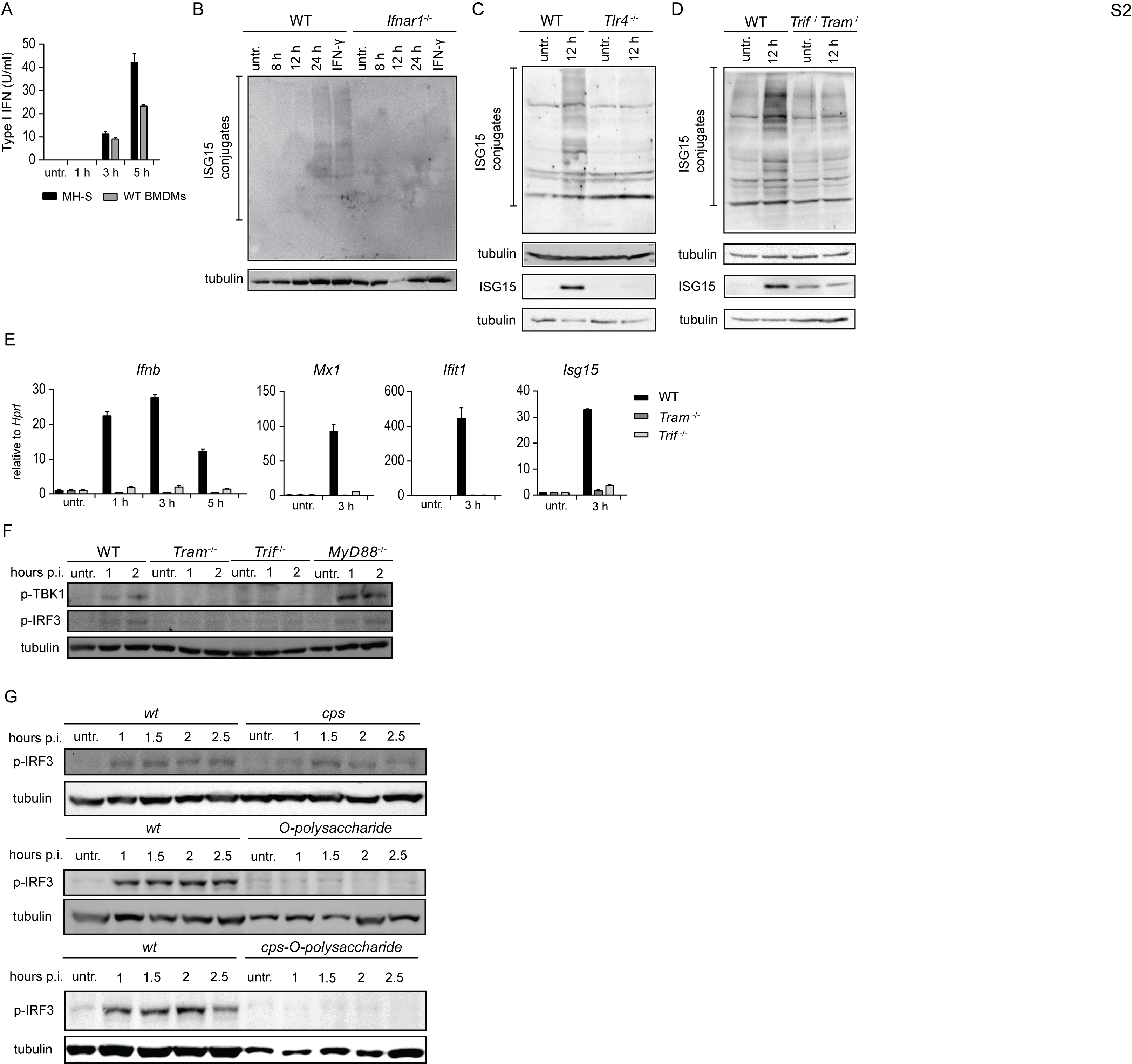

Supplement: S2 Fig — (A) Mouse alveolar macrophages (MH-S cell line) and BMDMs from WT mice were infected with K. pneumoniae (MOI = 70) for 1, 3 and 5 h, or left untreated, and type I IFN levels in the supernatant were determined. (n = 8). (B) BMDMs from WT and Ifnar1-/- mice were infected for indicated time points with K. pneumoniae, or treated with IFN-γ (for positive control) or left untreated. ISG15 conjugates and tubulin (loading control) were detected by in whole cell extracts by Western blotting. (C, D) BMDMs from WT and Tlr4-/- mice (C) or Tram-/-Trif-/- mice (D) were infected for 12 h with K. pneumoniae, or left untreated. ISG15 conjugates, ISG15 protein and tubulin (loading control) were detected in whole cell extracts by Western blotting. (E) WT, Tram-/- and Trif-/- BMDMs were infected as in (A) for indicated time points, or left untreated. Ifnb, Mx1, Ifit1 and Isg15 mRNA levels were determined by qPCR and normalized to Hprt. (F) WT, Tram-/-, Trif-/- and Myd88-/- BMDMs were infected as in (A) for indicated time points, or left untreated. p-TBK1, p-IRF3 and tubulin (loading control) were detected in whole cell extracts by Western blotting. (G) BMDMs were infected as in (A) with a cps, O-polysaccharide, and double cps-O-polysaccharide K. pneumoniae mutants for indicated time points, or left untreated. p-TBK1, p-IRF3 and tubulin (loading control) were detected in whole cell extracts by Western blotting. Statistical evaluation in (E): unpaired Student’s t test; error bars, mean ± SEM (n > 3). (TIF) [file ppat.1006696.s002.tif]

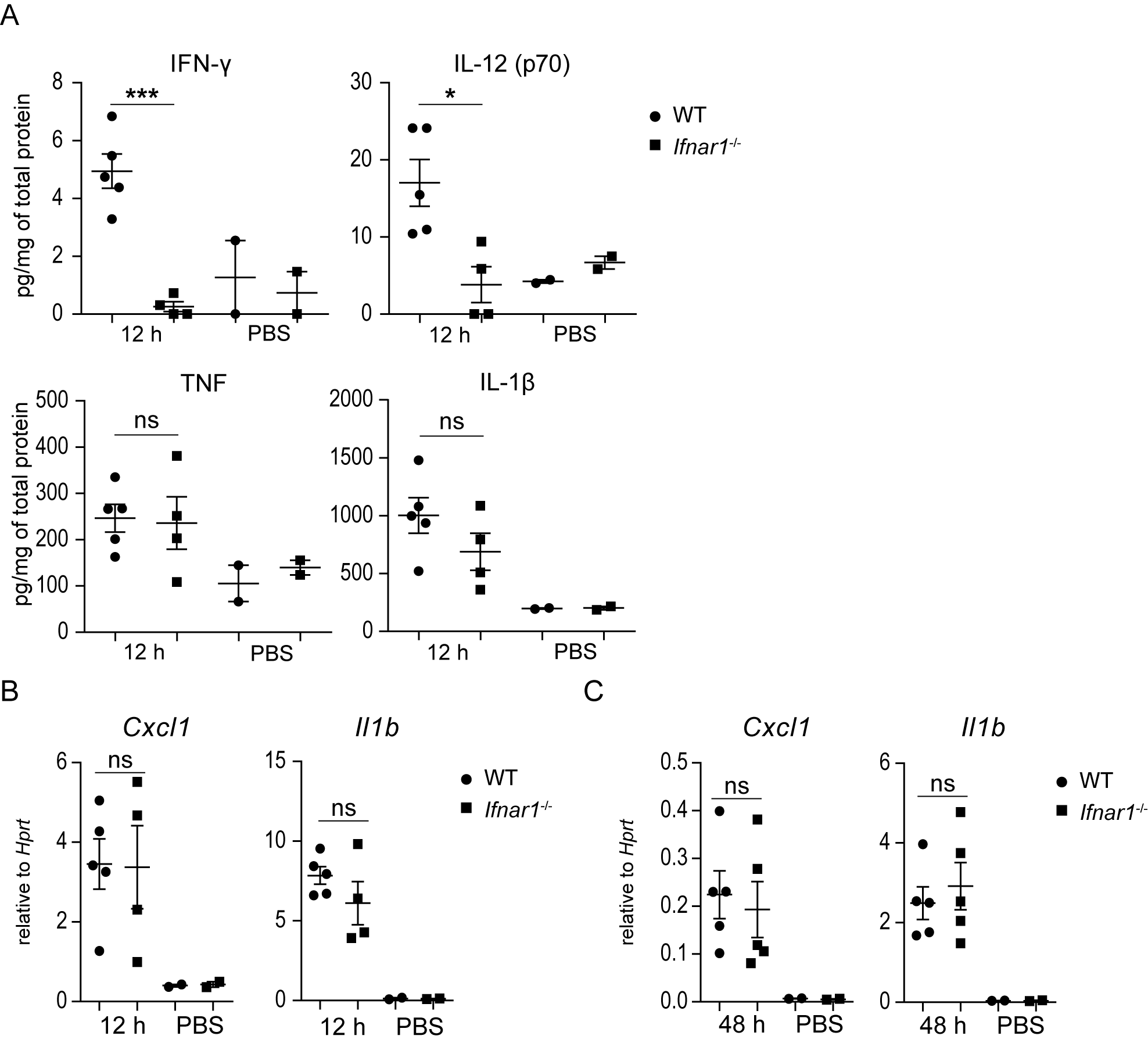

Supplement: S3 Fig — WT and Ifnar1-/- mice were infected intranasally (5 x 10^4^ CFU of K. pneumoniae) for 12 (A, B) or 48 (C) h, or treated with PBS. (A) IFN-γ, IL-12 (p70), TNF and IL-1β protein levels in lungs determined by ELISA. (B, C) Cxcl1 and Il1b mRNA levels determined by qPCR (normalized to Hprt). Statistical evaluation: unpaired Student’s t test; error bars, mean ± SEM (n > 3); *, P < 0.05; **, P < 0.01; ***, P < 0.001; ns, not significant. (TIF) [file ppat.1006696.s003.tif]

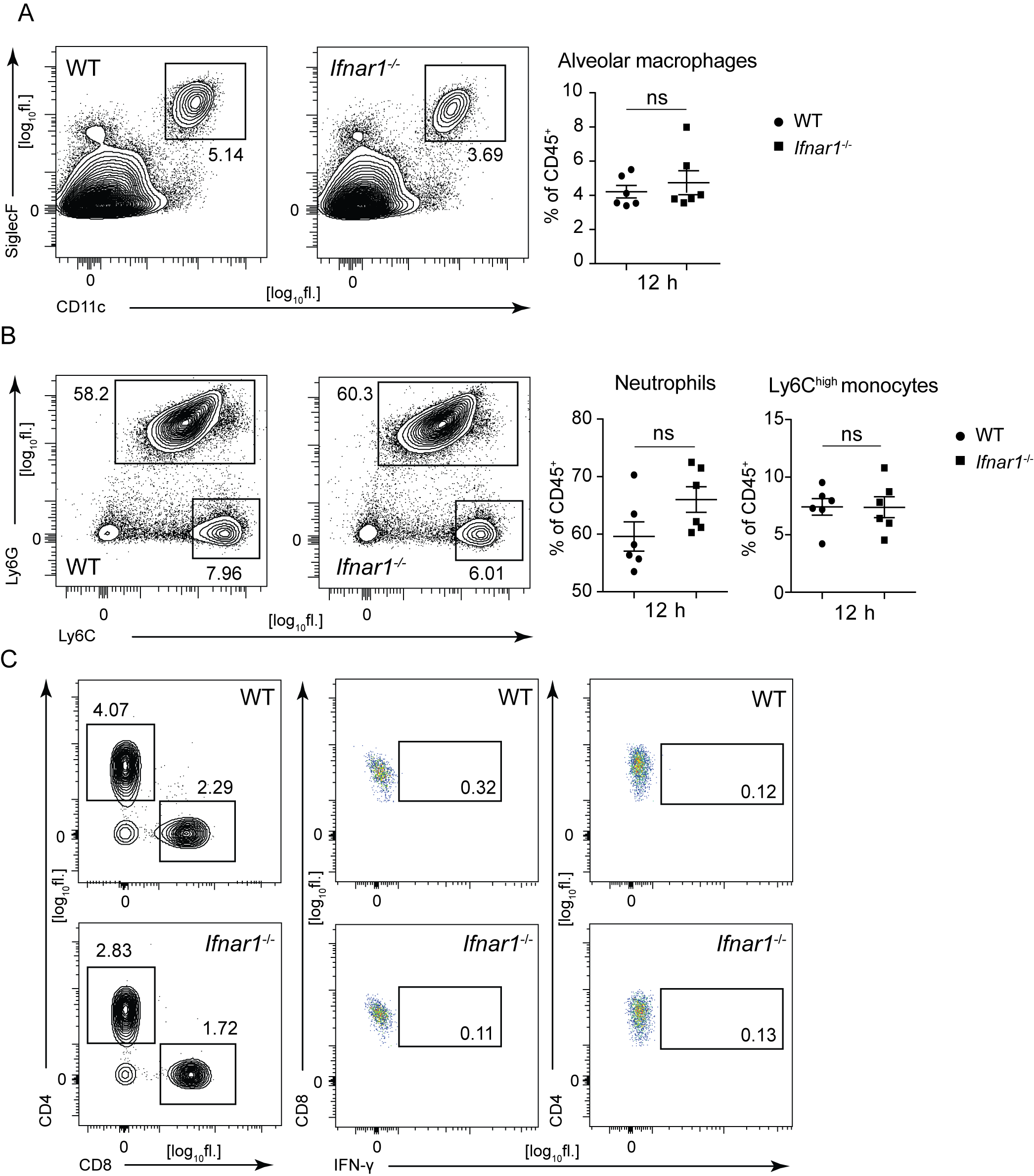

Supplement: S4 Fig — (A, B) Flow cytometry plots (representative experiments) and percentages of CD45+ cells for SiglecF+CD11chigh alveolar macrophages (A) and neutrophils (Cd11b+Ly6G+Ly6Cmed) with inflammatory monocytes (CD11b+Ly6G-Ly6Chigh) (B) are shown. (C) Representative flow cytometry plots of CD3+CD4+, CD3+CD8+, IFN-γ+ CD4 and IFN-γ+ CD8 T cells. Numbers indicate percentages in the outlined area of live CD45+ cells. (TIF) [file ppat.1006696.s004.tif]

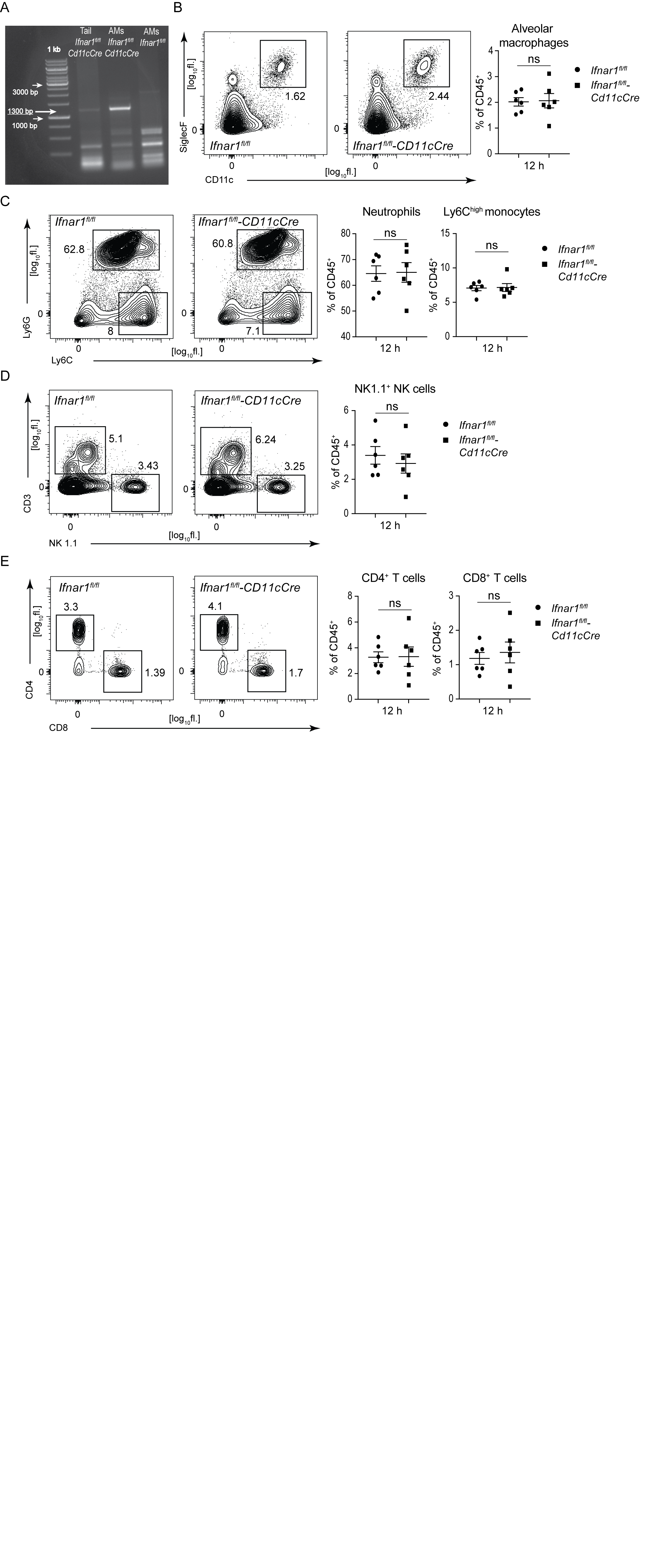

Supplement: S5 Fig — (A) PCR of genomic DNA isolated from alveolar macrophages (AMs) and tails of Ifnar1fl/fl-CD11cCre mice Ifnar1fl/fl mice. Indicated are 1000 and 3000 bp bands, as well as a deletion band (1300 bp). (B-D) Ifnar1fl/fl-CD11cCre and Ifnar1fl/fl mice (n = 6 per genotype) were infected intranasally (5 x 10^4^ CFU of K. pneumoniae) for 12 h, and immune cell subsets in lungs were analyzed by flow cytometry. Representative flow cytometry plots of alveolar macrophages (SiglecF+CD11chigh) (B, left panels), neutrophils (Cd11b+Ly6G+Ly6Cmed) and inflammatory monocytes (CD11b+Ly6G-Ly6Chigh) (C, left panels), NK cells (CD3-NK1.1+) (D, left panels), CD4 T cells (CD3+CD4+) and CD8 T cells (CD3+CD8+) (E, left panels) are shown. Numbers in the right panels indicate percentages of individual immune cell subsets in live CD45+ cells. Statistical evaluation: unpaired Student’s t test; error bars, mean ± SEM; ns, not significant. (TIF) [file ppat.1006696.s005.tif]

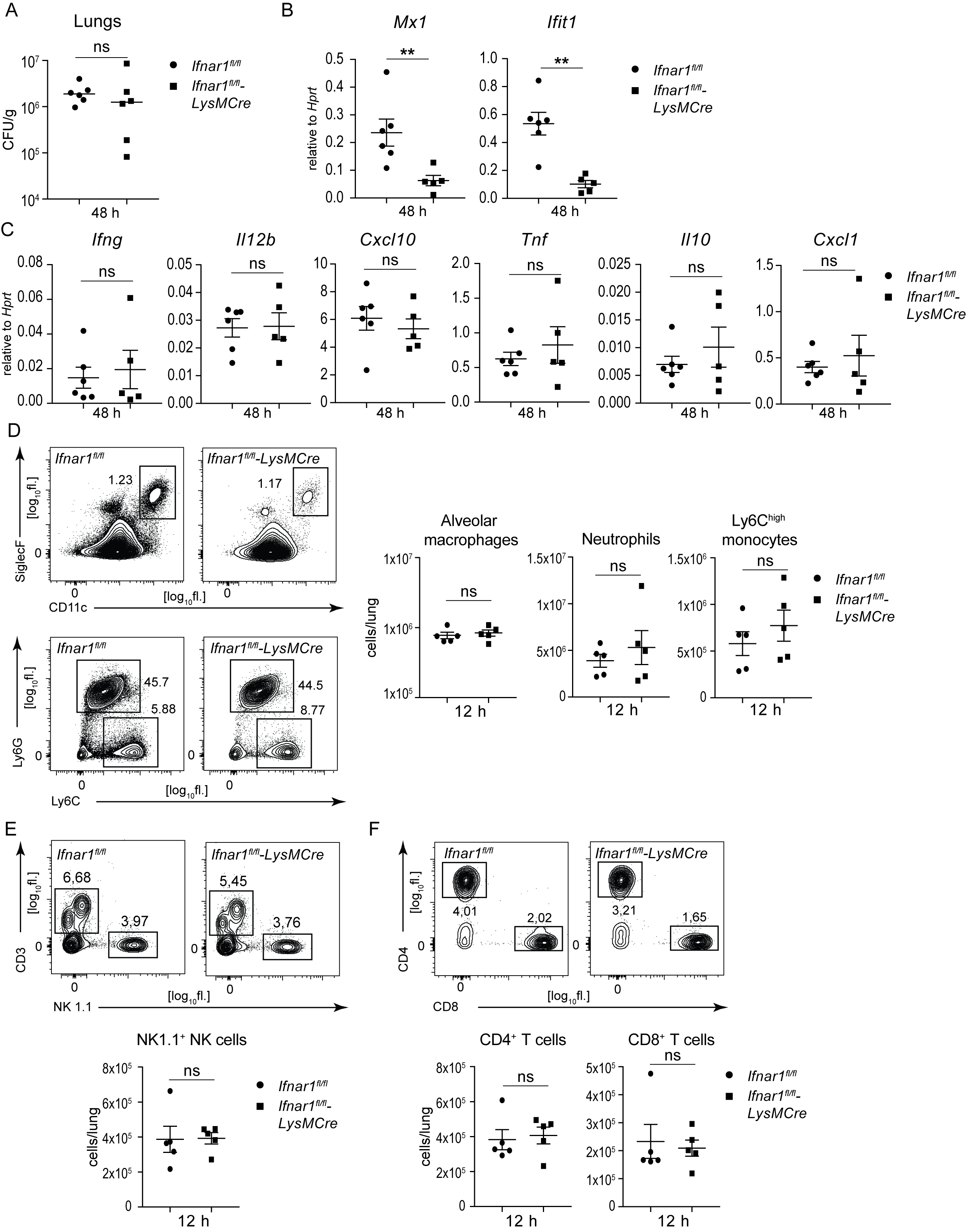

Supplement: S6 Fig — (A) Ifnar1fl/fl-LysMCre and Ifnar1fl/fl mice (n = 6 per genotype) were infected intranasally (5 x 10^4^ CFU of K. pneumoniae) for 48 h, and bacterial loads in lungs were determined. Statistical evaluation: Mann-Whitney test; ns, not significant. (B, C) Ifnar1fl/fl-LysMCre and Ifnar1fl/fl mice (n = 5 and 6, respectively) were infected as in (A). RNA was isolated from lungs and analyzed for expression of Mx1 and Ifit1 (B) as well as Ifng, Il12b, Cxcl10, Tnf, Il10 and Cxcl1 (C). Statistical evaluation: unpaired Student’s t test; error bars, mean ± SEM; **, P < 0.01; ns, not significant. (D-F) Ifnar1fl/fl-LysMCre and Ifnar1fl/fl mice (n = 5 per genotype) were infected as in (A). Immune cell subsets in lungs were analyzed by flow cytometry. Representative flow cytometry plots of alveolar macrophages (SiglecF+CD11chigh), neutrophils (Cd11b+Ly6G+Ly6Cmed) and inflammatory monocytes (CD11b+Ly6G-Ly6Chigh) (D, left panels), NK cells (CD3-NK1.1+) (E, upper panel), CD4 T cells (CD3+CD4+) and CD8 T cells (CD3+CD8+) (F, upper panels) are shown. Numbers in the right panels (D) and lower panels (E, F) indicate total numbers of individual immune cell subsets in lungs calculated from percentages of live CD45+ cells. Statistical evaluation: unpaired Student’s t test; error bars, mean ± SEM; ns, not significant. (TIF) [file ppat.1006696.s006.tif]

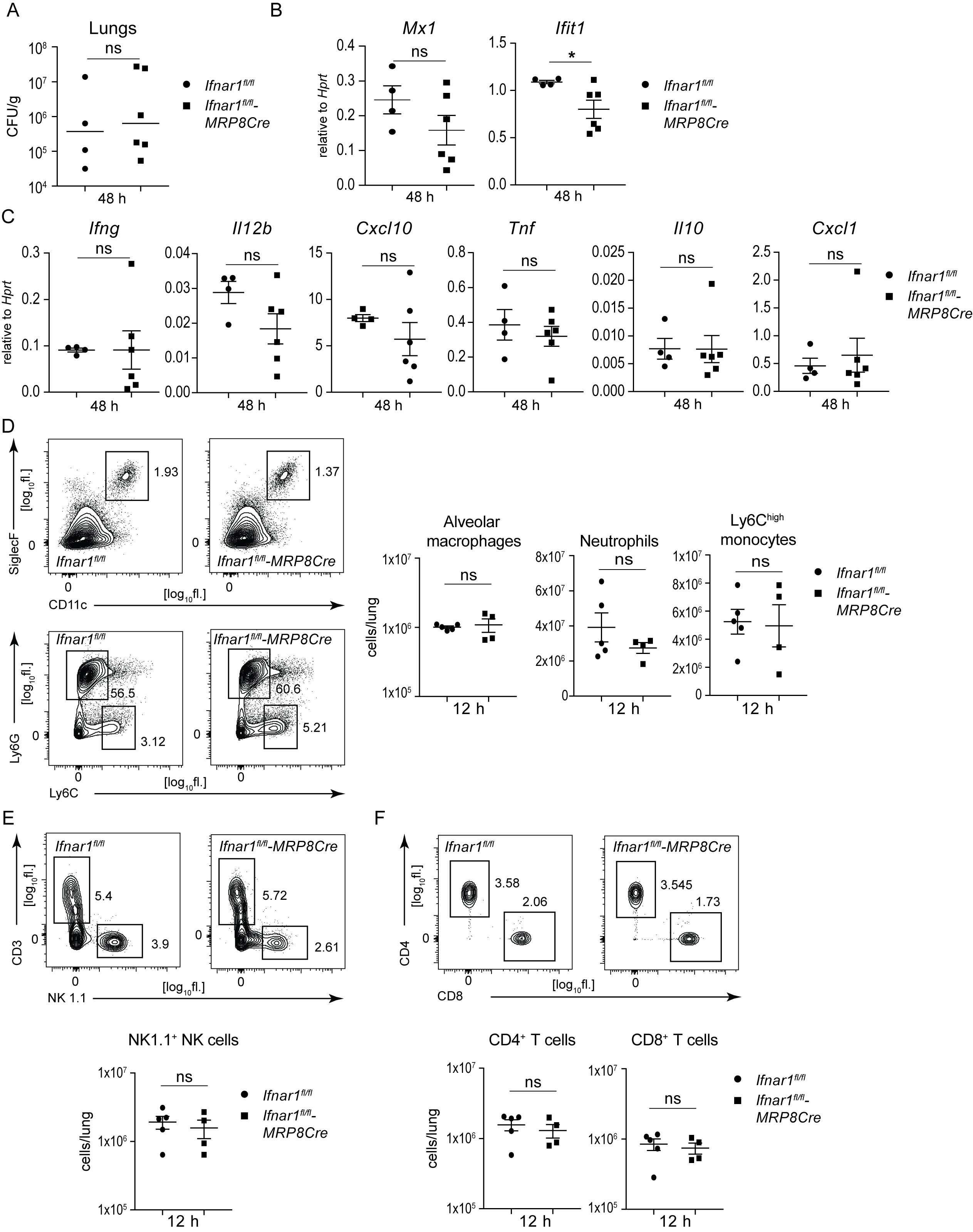

Supplement: S7 Fig — (A) Ifnar1fl/fl-MRP8Cre and Ifnar1fl/fl mice (n = 6 and 4, respectively) were infected intranasally (5 x 10^4^ CFU of K. pneumoniae) for 48 h, and bacterial loads in lungs were determined. Statistical evaluation: Mann-Whitney test; ns, not significant. (B, C) Ifnar1fl/fl-MRP8Cre and Ifnar1fl/fl mice (n = 6 and 4, respectively) were infected as in (A). RNA was isolated from lungs and analyzed for expression of Mx1 and Ifit1 (B) as well as Ifng, Il12b, Cxcl10, Tnf, Il10 and Cxcl1 (C). Statistical evaluation: unpaired Student’s t test; error bars, mean ± SEM; **, P < 0.01; ns, not significant. (D-F) Ifnar1fl/fl-MRP8Cre and Ifnar1fl/fl mice (n = 6 and 4, respectively) were infected as in (A). Immune cell subsets in lungs were analyzed by flow cytometry. Representative flow cytometry plots of alveolar macrophages (SiglecF+CD11chigh), neutrophils (Cd11b+Ly6G+Ly6Cmed) and inflammatory monocytes (CD11b+Ly6G-Ly6Chigh) (D, left panels), NK cells (CD3-NK1.1+) (E, upper panel), CD4 T cells (CD3+CD4+) and CD8 T cells (CD3+CD8+) (F, upper panels) are shown. Numbers in the right panels (D) and lower panels (E, F) indicate total numbers of individual immune cell subsets in lungs calculated from percentages of live CD45+ cells. Statistical evaluation: unpaired Student’s t test; error bars, mean ± SEM; ns, not significant. (TIF) [file ppat.1006696.s007.tif]

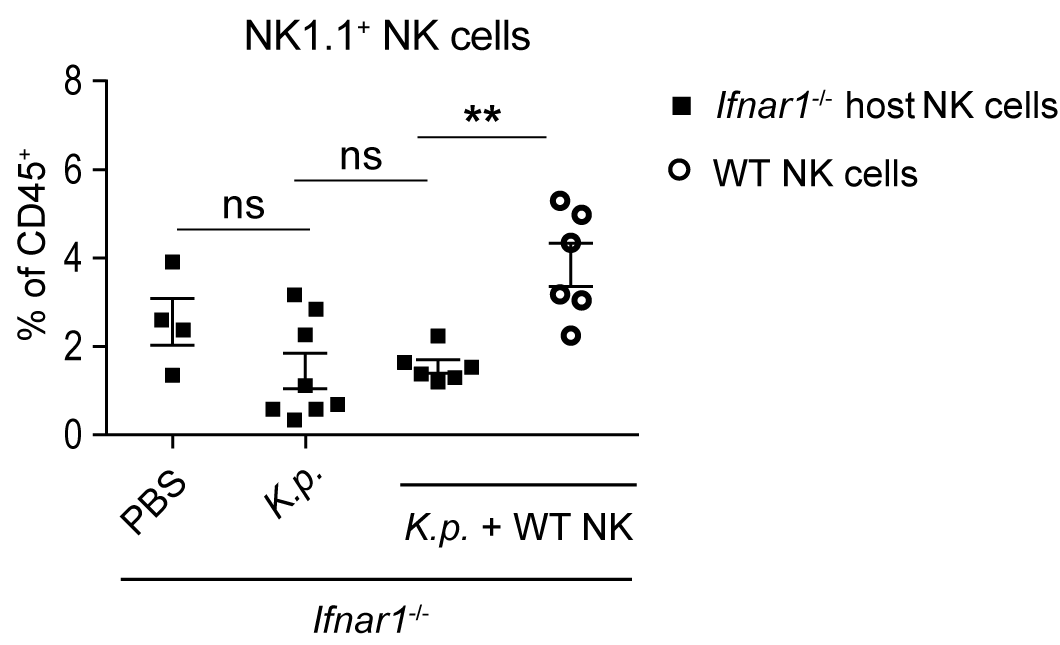

Supplement: S8 Fig — Ifnar1-/- mice were treated with PBS, infected intranasally (5 x 10^4^ CFU of K. pneumoniae) (K.p.), or given 1 x 10^6^ WT NK cells and infected intranasally (5 x 10^4^ CFU of K. pneumoniae) (K.p. + WT NK). Lungs were analyzed 24 h p.i. or treatment. Endogenous Ifnar1-/- NK cells (dot plot groups 1–3), as well as exogenous WT NK cells (dot plot group 4) detected by flow cytometry as CD3-NK1.1+ cells are shown in percent of CD45+ cells. Statistical evaluation: unpaired Student’s t test; error bars, mean ± SEM; **, P < 0.01; ns, not significant. (TIF) [file ppat.1006696.s008.tif]
